# Practical 4.7 V solid-state 18650 cylindrical lithium metal batteries with *in-situ* fabricated localized high-concentration polymer electrolytes

**DOI:** 10.1093/nsr/nwaf016

**Published:** 2025-01-17

**Authors:** Xingchen Song, Ruiqi Zhao, Jie Zhu, Jinping Zhang, Nuo Xu, Jie Liu, Yansong Liu, Hongtao Zhang, Yanfeng Ma, Chenxi Li, Yongsheng Chen

**Affiliations:** The Centre of Nanoscale Science and Technology and Key Laboratory of Functional Polymer Materials, Institute of Polymer Chemistry, College of Chemistry, Nankai University, Tianjin 300071, China; Renewable Energy Conversion and Storage Center (RECAST), Nankai University, Tianjin 300071, China; The Centre of Nanoscale Science and Technology and Key Laboratory of Functional Polymer Materials, Institute of Polymer Chemistry, College of Chemistry, Nankai University, Tianjin 300071, China; Renewable Energy Conversion and Storage Center (RECAST), Nankai University, Tianjin 300071, China; The Centre of Nanoscale Science and Technology and Key Laboratory of Functional Polymer Materials, Institute of Polymer Chemistry, College of Chemistry, Nankai University, Tianjin 300071, China; Renewable Energy Conversion and Storage Center (RECAST), Nankai University, Tianjin 300071, China; The Centre of Nanoscale Science and Technology and Key Laboratory of Functional Polymer Materials, Institute of Polymer Chemistry, College of Chemistry, Nankai University, Tianjin 300071, China; Renewable Energy Conversion and Storage Center (RECAST), Nankai University, Tianjin 300071, China; The Centre of Nanoscale Science and Technology and Key Laboratory of Functional Polymer Materials, Institute of Polymer Chemistry, College of Chemistry, Nankai University, Tianjin 300071, China; Renewable Energy Conversion and Storage Center (RECAST), Nankai University, Tianjin 300071, China; The Centre of Nanoscale Science and Technology and Key Laboratory of Functional Polymer Materials, Institute of Polymer Chemistry, College of Chemistry, Nankai University, Tianjin 300071, China; Renewable Energy Conversion and Storage Center (RECAST), Nankai University, Tianjin 300071, China; The Centre of Nanoscale Science and Technology and Key Laboratory of Functional Polymer Materials, Institute of Polymer Chemistry, College of Chemistry, Nankai University, Tianjin 300071, China; Renewable Energy Conversion and Storage Center (RECAST), Nankai University, Tianjin 300071, China; The Centre of Nanoscale Science and Technology and Key Laboratory of Functional Polymer Materials, Institute of Polymer Chemistry, College of Chemistry, Nankai University, Tianjin 300071, China; Renewable Energy Conversion and Storage Center (RECAST), Nankai University, Tianjin 300071, China; The Centre of Nanoscale Science and Technology and Key Laboratory of Functional Polymer Materials, Institute of Polymer Chemistry, College of Chemistry, Nankai University, Tianjin 300071, China; Renewable Energy Conversion and Storage Center (RECAST), Nankai University, Tianjin 300071, China; The Centre of Nanoscale Science and Technology and Key Laboratory of Functional Polymer Materials, Institute of Polymer Chemistry, College of Chemistry, Nankai University, Tianjin 300071, China; Renewable Energy Conversion and Storage Center (RECAST), Nankai University, Tianjin 300071, China; The Centre of Nanoscale Science and Technology and Key Laboratory of Functional Polymer Materials, Institute of Polymer Chemistry, College of Chemistry, Nankai University, Tianjin 300071, China; State Key Laboratory of Elemento-Organic Chemistry, Nankai University, Tianjin 300071, China; Renewable Energy Conversion and Storage Center (RECAST), Nankai University, Tianjin 300071, China

**Keywords:** lithium metal battery, solvation structure, gel polymer electrolyte, long cycle-life, high voltage

## Abstract

*In-situ* fabricated gel polymer electrolytes (GPEs), characterized with superior interfacial properties and large-scale processibility, represent a promising electrolyte system for high-performance lithium metal batteries (LMBs). Herein, we propose an *in-situ* fabricated high-voltage GPE featuring a localized high-concentration solvation structure (LHCE-GPE). This tailored special solvation structure within a polymer matrix promotes the formation of an electrochemically robust electrode–electrolyte interphase. Furthermore, employing LHCE-GPE, Li||Li_1.2_Ni_0.13_Co_0.13_Mn_0.54_O_2_ cells operating at 4.8 V demonstrate a high specific capacity of 248 mAh g^−1^, and 4.5 V Li||LiNi_0.8_Co_0.1_Mn_0.1_O_2_ cells achieve a remarkable cycling stability over 1000 cycles. Significantly, our LHCE-GPE allows for the operation of practical solid-state 18650 cylindrical LMBs at 4.7 V and industrial Li-ion batteries at 4.6 V, achieving high energy densities of 250 and 283 Wh kg^−1^, respectively (excluding packaging), while also demonstrating robust safety during rigorous nail-penetration tests. Our LHCE-GPE design presents a practical and powerful strategy for realizing solid-state LMBs with high energy density and high safety.

## Teaser

An in-situ fabricated gel polymer electrolyte, featuring a localized high concentration solvation structure, was fabricated for high-voltage lithium metal batteries, achieving ultra-long cycling stability and industry-level safety.

## INTRODUCTION

Lithium metal batteries (LMBs) are considered to be a highly promising candidate for next-generation rechargeable battery technologies due to their potential for significantly higher energy density, attributed to the high specific capacity (3860 mAh g^−1^) and low redox potential (−3.04 V vs. the standard hydrogen electrode) of the lithium metal anode (LMA) [1–3]. To further enhance the energy density of LMBs, high-voltage and high-capacity cathodes such as nickel-rich layered LiNi*_x_*Co*_y_*Mn_(1−_*_x_*_−_*_y_*_)_O_2_ (NCM) and Li-rich layered Li_1.2_Ni_0.13_Co_0.13_Mn_0.54_O_2_ (LNCMO) have been extensively investigated [4–7]. However, traditional organic liquid electrolytes are prone to uncontrollable side reactions with the highly reactive LMA, leading to continuous electrolyte decomposition loss, dendrite growth and irreversible capacity loss [8,9]. Moreover, the volatility and flammability of these liquid electrolytes pose severe safety risks, particularly in the event of short circuits, making them unsuitable for large-scale LMB applications [10,11].

Solid-state electrolytes (SSEs) have emerged as a promising solution to address these challenges, offering enhanced safety and stability compared to liquid electrolytes [12–16]. Among various SSEs, solid polymer electrolytes (SPEs), characterized by their processability and suitability for large-scale production, are considered to be one of the most promising systems to fully realize the potential of practical LMBs [17]. Recent advancements in polymer electrolyte design have focused on developing local high-concentration electrolyte (LHCE) structures. Wang *et al.* and Han *et al.* have pioneered an innovative approach using fluorinated polymers to regulate the solvation structure of lithium salts in residual solvents, resulting in films with excellent stability for both LMAs and high-voltage cathodes [18,19]. In addition, *in-situ* solidified gel polymer electrolytes (GPEs) have shown promise in achieving good electrode–electrolyte interfacial contact and enhanced Li-ion transport while remaining compatible with existing Li-ion battery manufacturing processes [20–25]. However, incorporating the LHCE structure into *in-situ* polymerized GPEs presents significant challenges, primarily due to solubility issues associated with fluorinated polymers and phase separation problems caused by interactions between polar solvents and the polymer matrix [26]. Overcoming these obstacles is crucial for realizing the full potential of LHCE-structured GPEs in advanced battery systems.

Furthermore, most of the studies on polymer-based electrolytes have primarily focused on laboratory-scale, small capacity coin and pouch cells, which still present a significant gap between experimental research and industrial-level practical batteries [22,27–29]. To bridge this gap and accelerate the development of high-energy–density, long-lifespan, and safe industrial-grade batteries, it is imperative to assess the performance of these electrolytes under near-industrial conditions. The widely used industrial-level 18650 cylindrical battery format presents an ideal platform for conducting such studies.

In this work, we designed and *in-situ* fabricated a high-voltage gel polymer electrolyte with a localized high-concentration solvation structure (LHCE-GPE), which exhibited superior oxidation stability up to 4.95 V and high ionic conductivity of 2.8 mS cm^−1^ at room temperature. The tailored special solvation structure of the solid-state polymer electrolyte facilitates the formation of an inorganic-rich interface layer, which effectively suppresses the growth of lithium dendrites and maintains the stability of the cathode structure under high voltage. By employing our LHCE-GPE system, we achieved impressive energy densities of 250 Wh kg^−1^ and 283 Wh kg^−1^ (excluding packaging) for solid-state 18650 cylindrical LMBs at 4.7 V and industrial lithium-ion batteries (LIBs) at 4.6 V, respectively. Furthermore, when the cut-off voltage was increased to 4.8 V, the LHCE-GPE enables Li||LNCMO cells to achieve a high specific capacity of 248 mAh g^−1^ at 0.5 C over 150 cycles. Moreover, Li||LiNi_0.8_Co_0.1_Mn_0.1_O_2_ (NCM811) cells using LHCE-GPE demonstrate remarkable cycling stability, enduring an ultra-long cycle life of 1000 cycles at 4.5 V and even a prolong life-span of 2000 cycles at 4.3 V, respectively, which are among the best results ever reported for solid polymer-based LMBs under comparable conditions (Table 1). Additionally, our designed LHCE-GPE delivers several other advantages including compatibility with various cathodes, superior low-temperature performance, scalable preparation processes and excellent safety features, which underscore its significant potential in high-performance practical LMBs.

**Table 1.** Comparison of the cycling performance of polymer electrolyte-based Li||NCM811 batteries with different cutoff voltages in previous work.

				Specific capacity	Number of	Capacity	
Polymer	Cathode material	Cutoff voltage	Rate	(mAh g^−1^)	cycles	retention	Ref
LiTFSI + G_4_ + TTE + PTEGDMA	Li||NCM811	4.5 V	1 C	200.4	1000	60%	This work
		4.3 V	2 C	179.2	1000	73%	
					2000	53%	
LiFSI + PVDF-LPPO	Li||NCM811	4.5 V	1 C	∼150	1000	61%	[50]
		4.3 V		∼140	1550	71%	
LiFSI + PDOX	Li||NCM811	4.5 V	1 C	193.5	100	84%	[51]
LiFSI + DME + PVDF-HFP + PTFEP	Li||NCM811	4.5 V	0.5 C	207	400	80%	[19]
LiTFSI + DOL-DME + PLE + PAN	Li||NCM811	4.4 V	1 C	154.1	500	43%	[52]
LiHFDF + LiTFSI-PEO	Li||NCM811	4.4 V	0.2 C	133.7	200	86%	[53]
LiTFSI + DEE + SFE + PETEA + PBCPN	Li||NCM811	4.4 V	0.5 C	200	500	75%	[54]
LiTFSI + PEGDME + PVDF-HFP	Li||NCM811	4.3 V	0.5 C	164.7	1000	65%	[55]
LiTFSI + SL + BN	Li||NCM811	4.3 V	0.2 C	182.4	300	90%	[56]
LiTFSI + PDMS-g-(PPEGMEMA-r-SPSS	Li||NCM811	4.3 V	0.5 C	162.2	200	72%	[57]
LiPF6 + FEC + FEMC + poly(FP + VC)	Li||NCM811	4.3 V	1 C	181.8	1200	81%	[58]

## RESULTS AND DISCUSSION

### Preparation and characterization of LHCE-GPE

**Figure 1.**
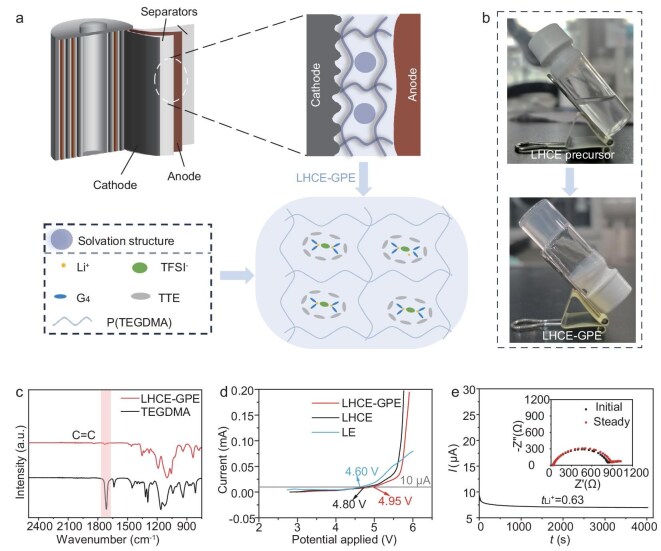
Preparation and characterization of LHCE-GPE. (a) Schematic illustration of the designed *in-situ* LHCE-GPE in an assembled cell (top) and the proposed solvation structure within LHCE-GPE (down). (b) Optical images of LHCE precursor (before) and LHCE-GPE (after polymerization). (c) FTIR spectra of TEGDMA monomer and LHCE-GPE. (d) Linear voltammetry curves of LHCE-GPE, LHCE and LE. (e) Chronoamperometric and Nyquist plots (inset) of LHCE-GPE at 25°C.

The synthesis of LHCE-GPE involved *in-situ* polymerization of a homogeneous precursor solution consisting of triethylene glycol dimethacrylate (TEGDMA) as the monomer and tailored LHCE-structured plasticizer at 60°C within an assembled cell (Fig. 1a). The plasticizer with LHCE-structure comprised a blend of tetraethylene glycol dimethyl ether (G_4_) solvent and 1,1,2,2‐tetrafluoroethyl-2,2,3,3‐tetrafluoropropyl ether (TTE) diluent (G_4_:TTE = 1:4 by mol), mixed with 1.2 M bis(trifluoromethanesulfonyl) imide (LiTFSI). The proposed solvation structure within the polymer matrix of LHCE-GPE, which is similar to widely reported LHCEs [30–32], *in-situ* ensures excellent electrode–electrolyte interface contact and improved electrode wettability, thereby reducing interface impedance and promoting uniform Li^+^ deposition. Figure 1b depicts the transformation of the colorless, transparent electrolyte precursor from liquid to a homogenous solid gel after polymerization.

Fourier transform infrared spectroscopy (FTIR) was employed to confirm the polymerization of the precursor solution. As shown in Fig. 1c, the C=C absorption peaks at 1636 cm^−1^, characteristic of TEGDMA, vanished after polymerization, indicating the successful synthesis of LHCE-GPE. Furthermore, the electrochemical stability of electrolytes was analyzed by linear sweep voltammetry (LSV) measurement. As shown in Fig. 1d, LHCE-GPE exhibits a high oxidation potential of up to 4.95 V, comparable to that (4.80 V) of the control LHCE (liquid electrolyte, without polymer matrix), and significantly exceeds that (4.65 V) of the commercial liquid electrolyte (LE, 1 M LiPF_6_ in ethylene carbonate:dimethyl carbonate = 1:1 vol%), attributing to the tailored LHCE structure. This high oxidation stability of LHCE-GPE, confirmed by electrochemical float analysis (EFA, >4.9 V, Fig. S1), demonstrates that its broad electrochemical stability window can accommodate the requirements of high-voltage cathodes such as nickel-rich layered NCM811 and layered Li-rich LNCMO cathodes, even at high charged cut-off voltages of 4.6 and 4.8 V, respectively. The ionic conductivities of the LHCE-GPE were subsequently evaluated by electrochemical impedance spectroscopy (EIS) measurements. As shown in Figs S2 and S3, the calculated ionic conductivity of the LHCE-GPE is 2.8 mS cm^−1^ at 25°C. In addition, by fitting the temperature dependence of the ionic conductivities to the Arrhenius equation, LHCE-GPE displays a low activation energy of 13.80 kJ mol^−1^, which is beneficial for Li^+^ transport and ensures satisfactory rate performance. Furthermore, LHCE-GPE exhibits an elevated Li^+^ transference number (*t*_Li^+^_) of 0.63 as shown in Fig. 1e, which is higher than that of the control LHCE (0.25, Fig. S3b). This increase in *t*_Li^+^_ results from the synergistic effects of the coordination effect of carbonyl groups in the polymer backbone with Li^+^ and the promoted Li^+^ transport facilitated by the ethylene oxide (EO) fragments in the polymer skeleton, which is also consistent with the molecular dynamics (MD) simulation results [33,34]. Furthermore, the elevated *t*_Li^+^_ of LHCE-GPE not only mitigates concentration polarization but also promotes uniform Li deposition and inhibit Li dendrite formation [34,35]. Moreover, the stress-strain curves of both LHCE-GPE and the separator were measured to evaluate their mechanical properties. As shown in Fig. S4, the LHCE-GPE exhibits an elongation rate of ∼83%, whereas the Celgard 2400 separator shows a lower elongation rate of ∼25%. These comparative findings highlight the superior ductility of our polymer electrolyte, which is capable of suppressing the growth of lithium dendrites.

### Solvation structure of LHCE-GPE

**Figure 2.**
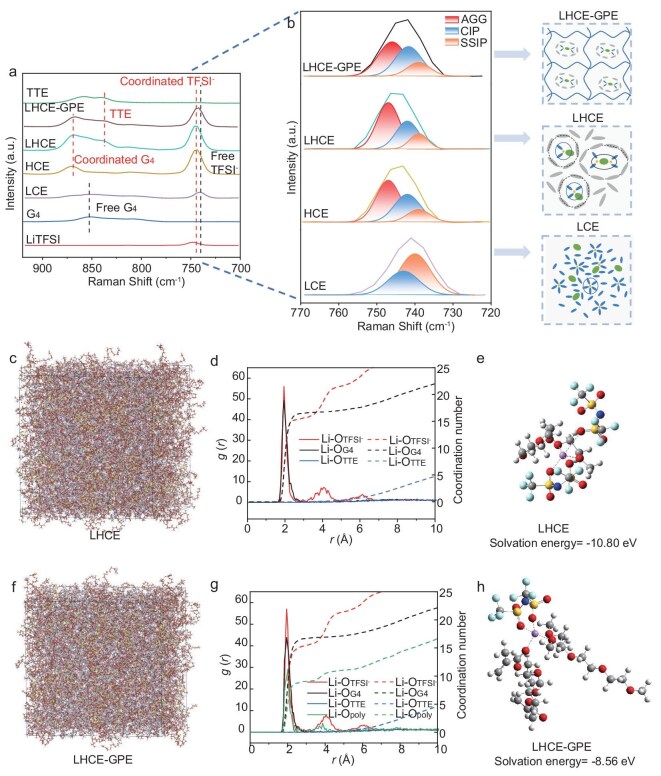
Solvation structures of LHCE-GPE and the control LHCE systems. Raman spectra and schematic diagrams of the Li^+^ coordination structures in LHCE-GPE (a) and LHCE (b) systems. Color code on the right side of Fig. 2b: Li^+^, yellow; G_4_, blue; TFSI^-^, green; TTE, cyan. Snapshots obtained from MD simulations of LHCE (c) and LHCE-GPE systems (f). Radial distribution functions in LHCE (d) and LHCE-GPE systems (g). The optimized Li^+^ solvation structures in the LHCE (e) and LHCE-GPE extracted from DFT calculation (h). Color code: C, grey; O, red; Li, purple; S, yellow; N, dark blue; F, cyan.

In addition to the polymer matrix, the evolution of solvation structures in GPEs plays a vital role in electrolyte systems, impacting on mitigating Li anode dendrite formation and expansion of the cathodic voltage range [36,37]. Raman spectroscopy, nuclear magnetic resonance (NMR) and MD analyses were carried out to investigate the solvation structures of LHCE-GPE and the control LHCE systems. As shown in Fig. 2a, LHCE-GPE displays a coordination Raman peak at 869 cm^−1^, indicating the coordination of G_4_ molecules with Li^+^ ions, which is similar to the coordination observed in LHCE and high-concentration electrolyte (HCE, 4.5 M LiTFSI in G_4_) systems. In contrast, the free G_4_ peak of 852 cm^−1^, typically observable in a low-concentration electrolyte (LCE:1.0 M LiTFSI in G_4_), is almost undetectable in the LHCE-GPE system. Furthermore, in the Raman vibration mode of TFSI^−^ (Fig. 2b), LHCE-GPE exhibits two peaks at 742 and 747 cm^−1^, corresponding to the intensities of contact ion pairs (CIPs) and cation–anion aggregates (AGGs), akin to those in the control LHCE and HCE systems, indicating the increased coordination probability of TFSI^−^ anions, markedly distinct from the reference LCE [38–40]. Thus, the absence of free G_4_ solvent and the anion-dominated solvation structure in LHCE-GPE contribute to its extended electrochemical window and enhanced electrode–electrolyte interfacial stability [41,42]. Based on these results, the proposed solvation configurations in LHCE-GPE, LHCE and LCE systems are depicted in Fig. 2b (right). Moreover, ^7^Li NMR was employed to investigate the solvation structure of LHCE-GPE. As illustrated in Fig. S5, the ^7^Li peak of LHCE-GPE exhibits a larger chemical shift (toward lower field) when compared to LHCE. This downfield shift suggests a reduction in the strength of chemical coordination around the Li^+^ ions within the LHCE-GPE system. Such a weakening can further promote the desolvation process of Li^+^ ions, thereby improving Li^+^ transport and increasing *t*_Li^+^_. The solvation structures of LHCE-GPE and the control liquid LHCE were further elucidated by MD simulations. The snapshots depicting the chemical structures of LHCE-GPE and LHCE are shown in Fig. 2c, f. In both electrolyte systems, cations (Li^+^), anions (TFSI^−^) and the solvating solvent (G_4_) were well dispersed to form the termed CIP and AGG structures. This Li^+^ solvation sheath in electrolytes was further supported by the corresponding radial distribution functions (RDFs, Fig. 2d and g) derived from MD simulations. In both LHCE-GPE and LHCE, TFSI^−^ anions enter the Li^+^ inner solvation sheath, presenting a high intensity of Li-O_TFSI_^−^ pairs at 1.95 Å. This solvation structure is suggested to be constructed of an inorganic species-rich and dense solid electrolyte interphase (SEI) on the LMA [43,44]. Based on these MD simulations (detailed in the Supplementary Information), the average coordination number of Li^+^ within the first solvation sheath of LHCE was calculated to be Li^+^(O_G4_)_3.09_(O_TFSI_^−^)_2.92_, where O_G4_ represents the oxygen atoms coordinated to Li^+^ from G_4_ molecules and O_TFSI_^−^ denotes the oxygen atoms coordinated to Li^+^ from TFSI^−^ anions. After introducing the poly(TEGDMA) matrix into LHCE, thereby forming the LHCE-GPE system, a fraction of the polymer was incorporated into the inner solvation sheath. This incorporation modifies the average coordination environment to Li^+^(O_G4_)_2.45_(O_TFSI_^−^)_2.24_(O_poly_)_1.31_, where O_poly_ represents the oxygen atoms coordinated to Li^+^ from the polymer. Notably, as shown in Fig. 2g, this introduction of the polymer does not significantly change the overall coordination environment of Li^+^, despite the redistribution of coordinating species. These solvation structures corroborate the conclusion obtained from the Raman analyses (Fig. 2a and b). The optimized solvation structures of Li^+^ in LHCE-GPE and LHCE, derived from the MD simulation, are illustrated in Fig. 2e, h, respectively. Compared to LHCE (−10.80 eV), the solvation energy of Li^+^ in LHCE-GPE is −8.56 eV (Fig. 2e and h), facilitating the desolvation and uniform deposition of Li^+^ [45]. Furthermore, as shown in Fig. S6, the desolvation energy of LHCE-GPE (62.58 kJ mol^−1^) is lower than that of the control LHCE (68.83 kJ mol^−1^), resulting in enhanced ionic conductivity and improved Li^+^ ion diffusion kinetics [35,46,47]. The aforementioned results are consistent with the NMR measurements above and in Fig. S5, indicating that the designed LHCE-GPE system promotes the Li^+^ desolvation and migration of Li^+^ during cycling. In addition, the exchange current density (*j*_0_) was investigated using Li||Li symmetric cells (Fig. S7). The value of *j*_0_ extracted from Tafel plots in LHCE-GPE (2.92 mA cm^−2^) was larger than that in LHCE (2.81 mA cm^−2^), indicating easier Li plating/stripping in the LHCE-GPE system.

### Performances of lithium metal half cells at high-voltage and wide temperature range

**Figure 3.**
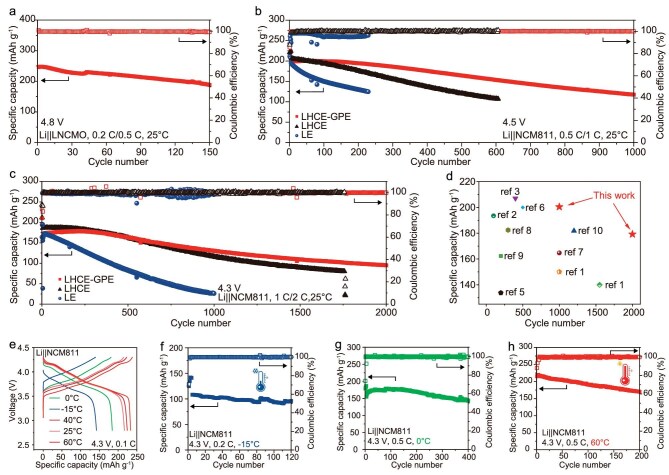
Electrochemical performances of LMBs with various cathodes at different operating voltages and temperatures. (a) Cycling performance of Li||LNCMO cells using LHCE-GPE at 0.5 C of 4.8 V. Cycling performances of Li||NCM811 cells with different electrolytes under the charge cut-off voltage of 4.5 V (b) and 4.3 V (c), respectively. (d) Performance comparison of solid-state full batteries. The related references are given in Table 1. (e) Charge-discharge profiles at the 3rd cycle of the Li|LHCE-GPE|NCM811 batteries at different temperatures. Cycling performance of the Li|LHCE-GPE|NCM811 batteries at −15°C (f), 0.5 C rate at 0°C (g) and 60°C at 0.5 C rate (h).

To demonstrate the applicability of LHCE-GPE as a promising polymer electrolyte, its electrochemical characteristics were evaluated in LMBs fabricated with various cathodes, including LNCMO (at 4.8 V), NCM811 (at 4.5 and 4.3 V), LiNi_0.6_Co_0.2_Mn_0.2_O_2_ (NCM622), LiCoO_2_ (LCO) and LiFePO_4_ (LFP). The excellent oxidative stability of LHCE-GPE, confirmed by LSV and EFA, enables stable cycling with LNCMO cathodes at a high voltage of 4.8 V (Fig. 3a and Fig. S8). Li||LNCMO cells utilizing LHCE-GPE deliver a high discharge specific capacity of 247.9 mAh g^−1^ (at 0.5 C) with a capacity retention of 76% after 150 cycles. In addition, Li|LHCE-GPE|LNCMO cells demonstrate a prolonged cycle life of 200 cycles at 4.7 V, along with a high discharge specific capacity of 237.2 mAh g^−1^ at 0.5 C (Fig. S9a and b).

To further verify the electrochemical performance of LHCE-GPE at elevated voltages, Li||NCM811 coin cells were assembled and evaluated at 4.5 V. Elevating the operating voltage to 4.5 V, beyond the commercial 4.3 V, enables the NCM811 cathode to deliver a higher specific capacity, aiming to achieve increased energy density [48,49]. As shown in Fig. 3b, 4.5 V Li|LHCE-GPE|NCM811 coin cells exhibit a high discharge capacity of 200.4 mAh g^−1^ at 1 C and an exceptionally long cycle life of 1000 cycles (60% capacity retention). In contrast, the control LHCE-based cells undergo significant capacity decay, retaining only 46% of their initial capacity after 700 cycles. On the contrary, 4.5 V Li||NCM811 batteries with commercial LE show rapid capacity decline, preserving 65% of their initial capacity after only 220 cycles. The overpotential data also reveal enhanced stability for the 4.5 V Li|LHCE-GPE|NCM811 cell over those using the control LHCE and LE, as depicted in Fig. S10. At an increased cut-off voltage of 4.6 V, Li|LHCE-GPE|NCM811 batteries achieve a higher discharge capacity of 217.7 mAh g^−1^, maintaining 69% capacity retention after 200 cycles (Fig. S11).

At a commonly used cut-off voltage of 4.3 V, Li|LHCE-GPE|NCM811 batteries deliver an ultra-long lifespan of 2000 cycles (53% capacity retention) with a high initial capacity of 179.2 mAh g^−1^ at a high discharging rate of 2 C (Fig. 3c and Fig. S12), which is far superior to the control liquid electrolytes (1700 and 1000 cycles of LHCE and LE, respectively). It also exhibits excellent discharge rate performance at 0.1, 0.2, 0.5, 1, 2, 5 and 10 C, corresponding to specific capacities of 209, 198, 185, 173, 154 and 128 mA h g^−1^ (1 C = 200 mA g^−1^). Upon transitioning from a high current density of 10 C to 0.5 C, the specific capacity remains unchanged, indicating that the LHCE-GPE–based cells can achieve full charge within 6 minutes at a high rate of 10 C, meeting the highly sought-after rapid charging requirements (Fig. S13). The cyclic voltammetry (CV) analysis of the Li|LHCE-GPE|NCM811 cell, as shown in Fig. S14, reveals well-defined oxidation/reduction peaks with consistent alignment across multiple cycles. These characteristics demonstrate minimal cell polarization and excellent reversibility for Li⁺ intercalation and deintercalation processes, indicating weak cell polarization and good reversibility for Li^+^ embedding/shedding. Notably, the electrochemical performance of our developed LHCE-GPE–based NCM811 batteries surpasses that of the currently reported polymer-based solid-state batteries at both 4.5 and 4.3 V (Fig. 3d and Table 1).

To validate the universality and compatibility of our solid LHCE-GPE electrolyte, Li||NCM622, Li||LCO and Li||LFP cells were also assembled and evaluated. As shown in Fig. S15, the Li||NCM622 and Li||LCO cells deliver a lifespan exceeding 1000 cycles at 4.3 V, with capacity retentions of 73% and 75%, respectively. Enabled by LHCE-GPE, the Li||LFP cell exhibits a high initial capacity of 132.3 mAh g^−1^ and maintains a capacity retention of 70% after 1800 cycles at 1 C. These results highlight LHCE-GPE's versatility with cutting-edge high-voltage cathodes and its compatibility with commercial battery assembly and manufacturing processes, underscoring its significant potential for practical applications.

Benefiting from its high Li^+^ conductivity and enhanced kinetics of Li^+^ desolvation, LHCE-GPE is expected to achieve excellent performance in LMBs under various temperature conditions. Indeed, Li|LHCE-GPE|NCM811 batteries exhibit outstanding performance across a broad temperature range, including at −15, 0, 25, 40 and 60°C, with specific capacities of 142.1, 184.5, 216.9, 224.6 and 233.6 mA h g^−1^, respectively (Fig. 3e). At a low temperature of −15°C, LHCE-GPE maintains a high ionic conductivity of 0.73 mS cm^−1^ (Fig. S16), enabling the Li||NCM811 cell to achieve a stable cycle life of 120 cycles with 89% capacity retention (Fig. 3f). At 0°C, a Li||NCM811 cell employing LHCE-GPE exhibits extended cycling stability, maintaining 81% capacity retention after 400 cycles (Fig. 3g). This superior low-temperature performance is attributed to the reduced desolvation energy of the polymer matrix in GPE. At a high temperature of 60°C, the Li|LHCE-GPE|NCM811 cell demonstrates a high initial capacity of 217.8 mAh g^−1^ and 78% capacity retention after 200 cycles at 0.5 C (Fig. 3h). The introduction of a polymer matrix stabilizes LHCE-GPE's structure and reduces solvent volatilization at elevated temperatures. These results demonstrate LHCE-GPE's adaptability to a wide range of temperature conditions.

### Cathode and anode interface chemistry and stability with LHCE-GPE electrolyte

**Figure 4.**
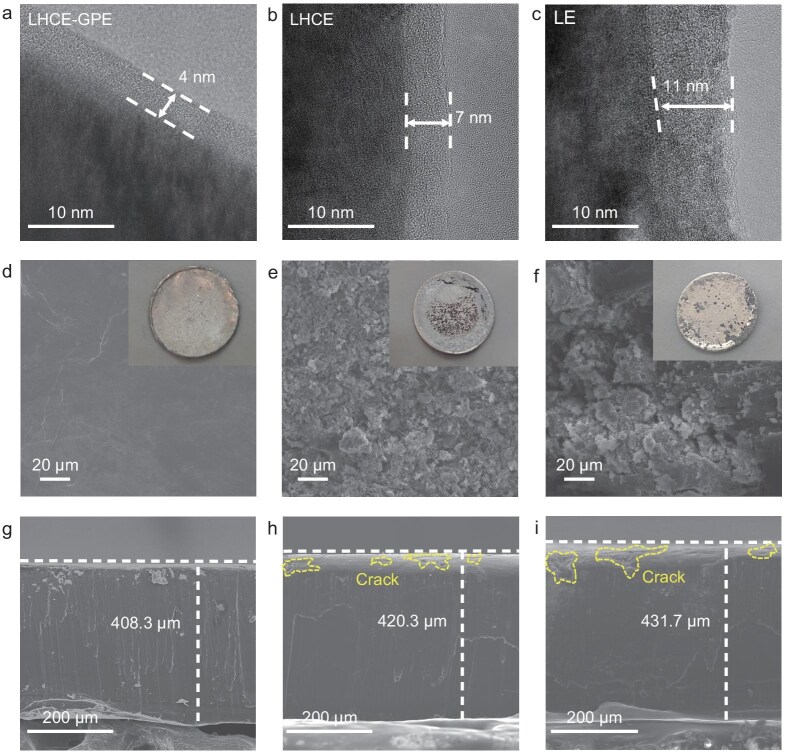
Characterizations of interface evolutions. TEM images of NCM811 cathodes after 100 cycles under a cut-off voltage of 4.3 V using LHCE-GPE (a), LHCE (b) and LE (c). Top-view and cross-sectional SEM images of Li metal anodes after 50 cycles with LHCE-GPE (d, g), LHCE (e, h) and LE (f, i). Insets: optical images of the cycled LMAs.

To elucidate the interface properties between the NCM811 cathode and LHCE-GPE, the NCM811 cathode was extracted from the Li||NCM811 coin cell after 100 cycles at 2 C and 4.3 V for subsequent analysis. The transmission electron microscopy (TEM) images in Fig. 4a, b reveal the formation of thin and uniform cathode electrolyte interphase (CEI) layers (∼4 and 7 nm) on the surfaces of the NCM811 cathodes cycled in LHCE-GPE and LHCE electrolytes, attributed to their special solvation structure. Furthermore, the layered structure of NCM811 cycled with LHCE-GPE was well-preserved, as evidenced by a clear lattice spacing of 0.2 nm corresponding to the (104) plane. This preservation of the crystal structure, as shown in Fig. S17, indicates the ability of LHCE-GPE to effectively stabilize cathode materials during high-voltage cycling [19]. In contrast, a thicker and non-uniform CEI layer (∼11 nm) was observed on the surface of the NCM811 cathode after cycling in commercial LE, as confirmed by the TEM image in Fig. 4c. To further investigate transition metal (TM) dissolution, the inductively coupled plasma-mass spectrometry (ICP-MS) was conducted on cycled-Li metal anodes. The result reveals significantly higher concentrations of Ni, Mn and Co in the LE and LHCE systems compared to LHCE-GPE, indicating that TM dissolution and crosstalk were effectively suppressed in the LHCE-GPE (Fig. S18). X-ray photoelectron spectroscopy (XPS) analyses were conducted on the surfaces of the NCM811 cathodes (after 100 cycles) to further characterize the components of the CEIs (Fig. S19). The peak at 399.7 eV was distinctly observed on the NCM811 interface, indicating the presence of Li-N rich inorganic components [59]. In contrast, high intensities of C–C (284.8 eV) and C–F (290.5 eV) peaks indicate undesired electrolyte decomposition on the surfaces of the NCM811 cathodes cycled with the control LHCE and LE electrolytes. The CEI component distribution was further investigated using time-of-flight secondary ion mass spectrometry (TOF-SIMS), as illustrated in Fig. S20. Abundant F^−^ and LiNO_3_^−^ signals were detected on the NCM811 surface, indicating that the CEI primarily consists of inorganic constituents generated from the decomposition of lithium salts. Organic components, represented by C_3_H_5_O^−^, were distributed in the upper layer of CEI, which contributes to the excellent cycle life and stable high cutoff voltage performance of the solid-state batteries. Moreover, scanning electron microscopy (SEM) images (Fig. S21) show that the NCM811 cathode, after 100 cycles in the polymer-based LHCE-GPE system, retains a spherical and smooth surface, with significant suppression or delay of the extensive intergranular cracking. In contrast, the NCM811 cathodes cycled in the control LHCE and LE liquid systems exhibit visible intergranular cracks, leading to increased exposure of fresh surfaces to the electrolyte.

Furthermore, to investigate the stability of the electrolyte in contact with lithium metal, Li||Li symmetric cells using LHCE-GPE and the control LHCE were evaluated at 0.2 and 0.5 mA cm^−2^, with a fixed capacity of 0.2 and 0.5 mAh cm^−2^ (Fig. S22). The cells incorporating the designed LHCE-GPE electrolyte demonstrated stable cycling performance over 1000 h. EIS was further conducted to verify the stability of the anode, as shown in Fig. S23. LHCE-GPE exhibits lower impedance, which further decreases during cycling compared to LHCE. These observations are consistent with the high cycling stability of the Li metal in the LHCE-GPE electrolyte. Moreover, critical current density (CCD) tests were conducted to evaluate the Li dendrite-suppression capabilities of LHCE-GPE and the control LHCE. Owing to its high ionic conductivity, LHCE-GPE exhibits a high CCD value of 3.9 mA cm^−2^, while the control LHCE without polymer matrix limits its CCD to 2.3 mA cm^−2^ (Fig. S24). Cross-sectional views and surface morphologies of cycled Li-metal anodes (after 50 cycles) with different electrolytes were also examined using SEM (Fig. 4d–i). For the cycled Li metal anode in the cell using LHCE-GPE, SEM images (Fig. 4d and g) exhibit a smooth surface and a dense deposition layer with minimal thickness increase (408.3 μm, compared to pristine 400 μm), demonstrating homogeneous Li deposition and retention of structural integrity. In contrast, as shown in Fig. 4e, f and h, i, cycled Li electrodes from the cells using the reference LHCE and LE show a whiskery structure, lots of cracks and increased thickness (420.3 and 431.7 µm, respectively), indicating continuous side reactions between electrolytes and the Li electrodes. Additionally, the optical photographs (Fig. 4d–f, insets) also confirm the smoother surface of Li foils in LHCE-GPE compared to control electrolytes (LHCE and LE), demonstrating the superior performance and safety features of LHCE-GPE.

As shown in Fig. S25, lithium deposited on the Cu foil in Li|LHCE-GPE|Cu cells exhibits a compact, large-particle morphology, which effectively alleviates parasitic reactions between LHCE-GPE and lithium metal. In contrast, lithium deposited in LHCE exhibits a loose and porous structure with numerous voids. Furthermore, by using the modified Aurbach method, the average Coulombic efficiency (CE) was calculated [60]. As shown in Fig. S26, the LHCE-GPE-based cell exhibits a 96.2% CE, which is higher than that (94.5%) of LHCE. Moreover, an analysis of the initial Li plating curves reveals that the nucleation overpotential of the LHCE-GPE is only 56.7 mV, smaller than the 83.0 mV of LHCE (Fig. S26b). This improvement is attributed to the construction of inorganic-rich SEI film in the LHCE-GPE system, which exhibits faster Li^+^ transport kinetics (Fig. S7) and contributes to the rapid and dense nucleation-growth of Li. XPS depth profiling was conducted to investigate the SEI compositions of the cycled Li metal in the three electrolytes. The N 1 s spectra of LHCE-GPE and LHCE electrolytes (Fig. S27) reveal significant signals for LiN_x_O_y_ (398.5 eV) [32], while the F 1 s spectrum disclosed the presence of LiF (685.2 eV) [61]. In addition, the chemical properties of the SEI on Li anodes after cycling were further analyzed by TOF-SIMS, as shown in Fig. S28. For the Li anode cycled in LHCE-GPE, the SEI mainly consists of inorganic species (Li_3_N^−^, F^−^ and LiS^−^), which is consistent with the XPS results [62]. This inorganic-rich composition suppresses side reactions and enhances performance of lithium plating/stripping [63,64].

### Practical application of LHCE-GPE in full coin cells, pouch cells and industrial-level 18650 cylindrical LMBs

**Figure 5.**
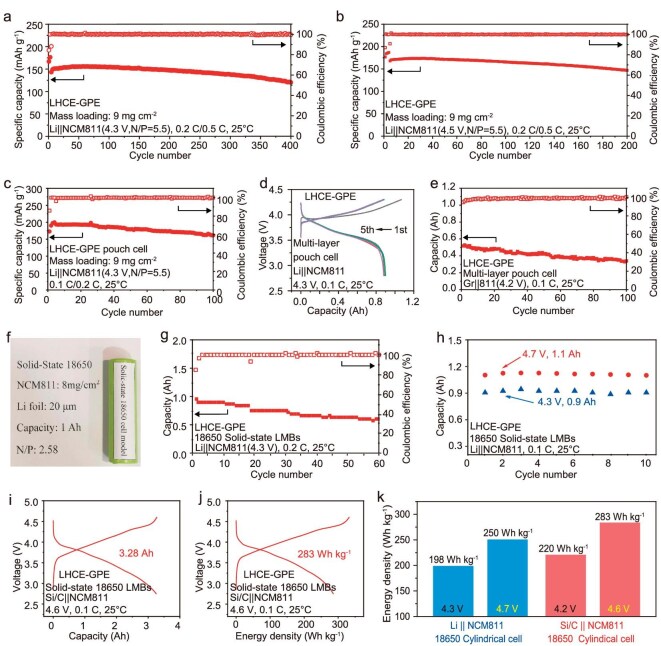
Practical applications of LHCE-GPE in full cells and industrial batteries. Cycling performances of Li||NCM811 coin full cells at 4.3 V (a) and 4.5 V (b), respectively. Cycling performances of single-layer (c) and multi-layer Li||NCM811 pouch cells (d). (e) Multi-layer graphite||NCM811 pouch cells. Optical images (f) and cycling performances (g) of 1 Ah solid-state 18650 Li||NCM811 LMBs. (h) Comparison of discharging capacity and energy density of solid-state 18650 cylindrical LMBs at 4.7 and 4.3 V. Voltage profiles (i) and energy density profiles (j) of solid-state 18650 cylindrical Si/C||NCM811 LIBs. (k) Energy density of Li||NCM811 and Si/C||NCM811 18650 cylindrical cells with different charging cutoff voltages at 0.1 C.

The practical full cells encompassing various battery types, such as coin-type, pouch and industrial 18650 cylindrical, were assembled to evaluate the true potential of LHCE-GPE. As shown in Fig. 5a, 5b and Fig. S29a, b, Li||NCM811 coin full cells with a high loading of 9 mg cm^−2^ (N/P ratio of 5.5) exhibit excellent cycling stability of 400 cycles at 4.3 V, retaining a capacity retention of 77%. To the best of our knowledge, this represents among the best cycling stabilities (400 cycles and 77% retention) of Li|| NCM811 full cells with thin Li foil and a high loading of cathode (9 mg cm^−1^) reported so far for polymer electrolyte-based batteries (Table S3). Upon increasing the operating voltage up to 4.5 V, Li|LHCE-GPE|NCM811 full cells (N/P ratio of 5.5) deliver an increased specific discharge capacity of 173.9 mAh g^−1^ with a capacity retention of 85% after 200 cycles. Moreover, graphite|LHCE-GPE|NCM811 full cells (N/P ratio of 1.3) realize prolonged cycling performance of 400 cycles with a capacity retention of 71% (Fig. S30).

To validate the practical application of LHCE-GPE, the cycling performance of Li| |NCM811 (high loading of 9 mg cm^−2^, N/P ratio of 5.5) single-layer pouch full cells were evaluated, delivering a retention rate of 83% after 100 cycles (Fig. 5c and Fig. S29c). Furthermore, the multi-layer Li|LHCE-GPE|NCM811 pouch cells, with an ultra-high cathode mass loading of 17.7 mg cm^−2^ (areal capacity of 3.54 mAh cm^−2^), exhibit a high capacity of 0.9 Ah and an energy density of 173 Wh kg^−1^ at 0.1 C. The initial five charge-discharge curves suggest the formation of an outstanding interface layer (Fig. 5d). Moreover, as shown in Fig. 5e and Fig. S29d, multi-layer griphite|LHCE-GPE|NCM811 pouch cells also display a stable cycling performance across 100 cycles.

In terms of industrial-level LIBs, the 18650 model is arguably the most important one. Therefore, it would be highly significant to design and study LMBs using this model as the platform. The *in-situ* LHCE-GPE electrolyte exhibits its compatibility with the industrial production process of the 18650 battery, suggesting that it has the potential to significantly enhance LMB technologies in alignment with established manufacturing processes. Thus, we fabricated practical, ampere-hour-level, solid-state 18650 cylindrical LMBs with a theoretical capacity of 1 Ah (NCM811 cathodes: 8 mg cm^−2^ and ultra-thin Li anode: 20 µm) via an *in-situ* polymerization process (Fig. 5f). Using our designed LHCE-GPE electrolyte, the solid-state 18650 cylindrical Li||NCM811 cells exhibit an initial capacity of 0.91 Ah at 4.3 V and maintain 60 stable cycles (Fig. 5g and h). When the working voltage increased to 4.7 V, due to the excellent oxidation stability of LHCE-GPE, 18650 cylindrical LMBs exhibit an enhanced capacity of 1.1 Ah and a high energy density of 250 Wh kg^−1^, which represents an increase of 26.3% compared to the energy density at 4.3 V (198 Wh kg^−1^, excluding package, more details in Table S1). Furthermore, the LHCE-GPE was also applied to industrial 18650 LIBs (cathode: NCM811; anode: Si/C). When operating at 4.6 V, the battery can provide a capacity of 3.28 Ah and an energy density of 283 Wh kg^−1^ (Fig. 5i and j), a 28.6% increase over the energy density of LIBs at 4.3 V (220 Wh kg^−1^, Table S2). As shown in Fig. 5k, our developed LHCE-GPE can significantly increase the energy density of practical batteries (26.3% and 28.6%, respectively) at high cutoff voltage. Additionally, Li_4_Ti_5_O_12_|LHCE-GPE|LiMn_2_O_4_ 18650 cylindrical LIBs also deliver a high capacity of 1.3 Ah and a long cycle life of 300 cycles (detailed in Fig. S31 and Table S4). Consequently, the exceptional oxidative stability of the well-designed LHCE-GPE enables a significant enhancement in energy density for practical, industrial-level batteries, illustrating its effectiveness and potential in modern battery technology.

### Safety tests of LHCE-GPE-based practical solid-state LMBs

**Figure 6.**
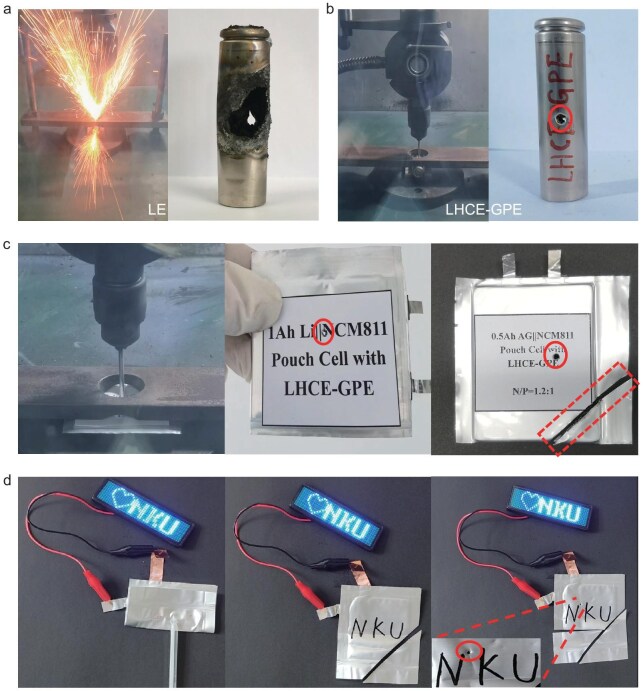
Safety tests of the solid-state polymer electrolyte in various 18650 cylindrical and pouch LMBs. Nail penetration tests of 18650 Li|LE|NCM811 cells (a), solid-state 18650 Li|LHCE-GPE|NCM811 cells (b) and multi-layer Li|LHCE-GPE|NCM811 pouch cells and multi-layer AG|LHCE-GPE|NCM811 pouch cells (c). (d) Safety test of Li||NMC811 pouch cells under various conditions including bending, cutting and punching.

Enhancing the safety of practical batteries is a primary objective in the development of solid-state batteries. High capacity (ampere-hour scale) pouch cells and cylindrical batteries incorporating LMA are susceptible to electrolyte leakage and potential ignition under mechanical abuse conditions, such as penetration and shearing, presenting significant safety risks in practical applications. Therefore, industry-standard nail penetration and cutting tests were conducted on pouch cells and 18650 cylindrical LMBs under a fully charged state of 4.3 V to investigate cell safety under extreme short-circuit conditions. As shown in Fig. 6a and Video S1, the reference 18650 cylindrical LMB with LE shows a violent explosion upon nail penetration. In stark contrast, the 18650 Li||NCM811 cylindrical cell using our designed LHCE-GPE electrolyte exhibits excellent safety, showing no electrolyte leakage or combustion during the nail penetration process, due to the solidified polymer electrolyte (Fig. 6b, Video S2). Moreover, as shown in Fig. 6c, the Ah-level LHCE-GPE–based solid-state pouch cells (with Li metal and graphite anode, respectively) also maintain their intact shape and exhibit no electrolyte leakage after nail penetration and cutting. Additionally, the Li|LHCE-GPE|NCM811 soft pouch cell can continue to power a light emitting diode (LED) screen with the NKU logo, even after undergoing bending, cutting and nail-penetrating (Fig. 6d). Consequently, our designed LHCE-GPE electrolyte system demonstrates its exceptional safety and adaptability under a wide range of harsh conditions, making it suitable for real-world applications.

## CONCLUSION

In summary, we have designed and fabricated LHCE-GPE via a tailoring solvation chemistry and *in-situ* polymerization strategy, demonstrating exceptional oxidation stability and high ionic conductivity. The specially tailored solvation structure within the polymer matrix promotes the formation of an electrochemically robust electrode–electrolyte interphase, facilitating uniform Li deposition and enhancing interfacial stability. As a result, Li|LHCE-GPE|LNCMO cells operating at 4.8 V exhibit a high specific capacity of 247.9 mAh g^−1^ with stable cycling over 150 cycles. Furthermore, Li|LHCE-GPE|NCM811 batteries achieve remarkable ultra-long lifespans of 1000 cycles at 4.5 V and 2000 cycles and 4.3 V, respectively, exhibiting the best performance as recorded so far. More importantly, our LHCE-GPE enables practical solid-state 18650 cylindrical LMBs to deliver a high energy density of 250 Wh kg^−1^ at 4.7 V, while industrial cylindrical LIBs achieve 283 Wh kg^−1^ at 4.6 V. These batteries also demonstrate outstanding safety toward rigorous mechanical abuse. Furthermore, the LHCE-GPE–based batteries exhibit excellent performance across a wide temperature range from −15 to 60°C, highlighting their potential for real-world applications. Thus, our localized high-concentration electrolyte concept for designing polymer electrolytes provides a promising pathway for high-performance, high-voltage and high-safety practical LMBs.

## Supplementary Material

nwaf016_Supplemental_Files
