# Supplementary material for: Practical 4.7 V solid-state 18650 cylindrical lithium metal batteries with in-situ fabricated localized high-concentration polymer electrolytes
Source: Natl Sci Rev. 2025 Jan 17;12(4):nwaf016. doi: 10.1093/nsr/nwaf016 (PMC11887857; doi:10.1093/nsr/nwaf016)
Supplement: nwaf016_Supplemental_Files [file nwaf016_supplemental_files.zip › 1403-Supporting Information.pdf]

Practical 4.7 V solid-state 18650 cylindrical lithium metal batteries with *in-situ* fabricated localized high-concentration polymer electrolytes

Xingchen Song[1,3], Ruiqi Zhao[1,3], Jie Zhu[1,3], Jinping Zhang[1,3], Nuo Xu[1,3], Jie Liu[1,3], Yansong Liu[1,3], Hongtao Zhang*[1,3], Yanfeng Ma[1,3], Chenxi Li[1,3], and Yongsheng Chen *[1,2,3]

[1]The Centre of Nanoscale Science and Technology and Key Laboratory of Functional Polymer Materials, Institute of Pomer Chemistry, College of Chemistry, Nankai University, Tianjin 300071, China

[2]State Key Laboratory of Elemento-Organic Chemistry, Nankai University, Tianjin, 300071, China

[3]Renewable Energy Conversion and Storage Center (RECAST), Nankai University, Tianjin 300071, China

Corresponding authors. E-mails: yschen99@nankai.edu.cn; htzhang@nankai.edu.cn

Table of contents

Section S1. Materials

Section S2. Synthesis of LHCE and LHCE-GPE

Section S3. Preparation of Electrodes and Fabrication of Batteries

Section S4. Characterizations and Electrochemical Measurements

Section S5. Computational Methods and Calculation

Section S6. Supplementary Figures and Tables

Section S7. Supplementary references

Section S1. Materials

Tetraethylene glycol dimethyl ether (G_4), triethylene glycol dimethacrylate (TEGDMA) and lituium difluorophosphate ($LiPO_2F_2$) were purchased from Macklin. Lithium bis(trifluoromethanesulfonyl)imide (LiTFSI), 1,1,2,2-tetrafluoroethyl-2,2,3,3-tetrafluoropropyl ether (TTE), fluoroethylene carbonate (FEC) were purchased from Duoduo chemical company in Suzhou. Azobisisoheptanenitrile (ABVN) was purchased from Energy Chemical.

Section S2. Synthesis of LHCE and LHCE-GPE

The preparation of the electrolyte was conducted in an Ar-filled glovebox. The low-concentration electrolyte (LCE) was prepared by dissolving 1.0 mol LiTFSI in 1.0 L G_4 solvent, the HCE was prepared by dissolving 4.5 mol LiTFSI in 1.0 L G_4. The LHCE was prepared by diluting the 4.5 M G_4-HCE with TTE at a G_4: TTE molar ratio of 1: 4, and then 0.5 wt% LiDFP and 0.5 wt% FEC were added into the organic solvents as the additives. 1.0 g LHCE was mixed with 0.04 g TEGDMA monomer and 0.4 mg ABVN to obtain gel electrolyte precursor, and then the precursor was *in-situ* polymerized at 60 °C for 24 h to obtain LHCE-GPE.

Section S3. Preparation of Electrodes and Fabrication of Batteries

Preparation of Electrodes: The cathodes and graphite anode were fabricated as follows. The cathode active materials (e.g. LNCMO, NCM811, NCM622, LCO, LFP), super P and poly (vinylidene fluoride) (PVDF) binder were mixed with a weight ratio of 8:1:1 via N-methyl-2-pyrrolide (NMP) as a solvent. It was then uniformly coated on the surface of the aluminum foil and dried in drying oven for 3 h at 60 °C, then 3 h in vacuum oven at 120 °C, 6 h in vacuum oven at 150 °C. The cathode was tailed a diameter of 10 mm and had an active material load of about 2 mg cm^{-2}, 9 mg cm^{-2}. The double-sided high load NCM811 cathodes (8 mg cm^{-2}) were purchased from Guangdong Canrd New Energy Technology Co., Ltd. and can be used after drying in a vacuum oven at 150 °C for 6 hours. The preparation process of graphite anode is the same as above, but the current collector is Cu foil and the

vacuum drying temperature is 80 °C. Ultra-thin Li foil (50 μm and 20 μm) with Cu current collector was purchased from China Energy Lithium Co., Ltd..

Fabrication of Batteries: LIR2032 type coin cell or pouch cell were assembled with the above cathodes, Li anode, Celgard 2400 and precursor solution (50 μL in coin cell and 2 mL in pouch cell) in the Ar-filled glove box (Mikrouna, $[O_2] < 0.01$ ppm, $[H_2O] < 0.01$ ppm). Then the batteries were kept in a 60 °C oven for 24 h.

The multi-layer Li||NCM811 and Gr||NCM811 pouch cells without electrolyte were purchased from Guangdong Canrd New Energy Technology Co., Ltd. and LiFun Technology, respectively. 6 g electrolyte was added into each cell. After that, the pouch cells were heated at 60 °C for 24 h. The $LiMn_2O_4$||$Li_4Ti_5O_{12}$ and Si/C||NCM811 18650 cylindrical cells without electrolyte were purchased from Tianjin PlanNano Company and Jiangxi Far East Battery co., LTD and dried before use. And then precursor was injected. After that, the cylindrical cell was heated at 60 °C for 24 h. All batteries underwent 3 pre-cycles at 0.1 C charge/discharge for activation before testing. For the nail penetration tests, the cells were first charged to 100% stat-of-charge, and then a nail was penetrated into the center of cells at a constant speed.

Section S4. Characterizations and Electrochemical Measurements

Characterizations: Fourier transform infrared spectroscopy (FT-IR) measurements were carried on Thermo Scientific Nicolet iS50 FT-IR spectrometer in the wavelength range of 400-4000 cm^{-1} through the attenuated total reflection (ATR) model (solid sample with KBr pellets). Raman spectroscopic measurement was performed by WiTecalpha300R confocal Raman microscope. The Liquid NMR of LHCE and LHCE-GPE were carried on a Bruker AV 600 spectrometer at 600 MHz (^7Li NMR) by dissolving LHCE and LHCE-GPE in chloroform-d. The Solid-state NMR of LHCE-GPE was carried on a Bruker AVANCE NEO 400 MHz wide-bore (89 mm) spectrometer with ^7Li frequency of 155.66 Hz. The electrode sample (Li anode or NMC811 cathode) was obtained by disassembling the battery, stripping polymer electrolyte from the surface, and washing the electrode with DMC more than three times to remove residual electrolytes before characterization. The morphology of the

electrode was observed by the PhenomPro SEM system. The X-ray photoelectron spectroscopy (XPS) spectra was collected on Thermo Scientific K-Alpha$^+$ with monochromatic 72 W Al Kα radiation. The transmission electron microscope was measured on JEOL JEM-F200. The time-of-flight secondary ion mass spectrometry (TOF-SIMS) was performed on a TOF.SIMS 5-100 spectrometer equipped with a 1 keV Cs^+ ion beam for sputtering. Samples were analyzed using a 30 keV Bi^+ ion beam over an area of 50 × 50 μm^2. Prior to ICP-MS (Agilent ICP-MS 7700S) analysis, the samples for cycled-Li metal anodes were prepared using a microwave reaction in a solution consisting of 25 vol% of concentrated nitric acid and 75 vol% of concentrated hydrochloric acid. Transmission electron microscopy (TEM) was taken on an FEI Tecnai F30 High-Resolution Transmission Electron Microscope with the operating voltage at 200 kV. The stress-strain test of the sample was performed on a UTM6103 mechanical testing instrument (Shenzhen Suns Technology Stock Co., LTD., China) with a tensile speed of 50 mm min^{-1}.

Electrochemical Measurements: The electrochemical measurements of LHCE-GPE were carried out by *in-situ* fabrication. All the measurements were carried out at 25 °C if not mentioned. The electrochemical floating analysis and the electrochemical performance tests were performed using a LAND battery testing system (Land CT2001A model, Wuhan LAND Electronics. Ltd.) and NEWARE (CT-4008Tn-5V6A-S1-F) instruments. The cycle and rate performance were measured after activation under 0.1C for 3 cycles. linear sweep voltammetry (LSV), Electrochemical impedance spectroscopy (EIS) tests and Cyclic voltammetry (CV) were conducted by using a Vionic (Metrohm). The LSV test was performed at the scan rate of 1mV s^{-1} from 2-6 V vs Li^+/Li at room temperature. EIS was measured from 10^6 HZ to 10^{-1} HZ with an alternating voltage of 5 mV. CV analysis was conducted in the potential window of 2.8-4.3 V with scan rate of 0.1 mV s^{-1}. The ionic conductivities of the LHCE-GPE were measured by assembling coin cells though *in-situ* polymerization procedure with two pieces of stainless steels separated by a thin insulating ring. The ionic conductivity (σ) can be calculated by the following equation (1):

$$\sigma=L/RS \tag{1}$$

where L and S are the thickness and the hole area of the insulating ring, and R is the bulk resistance of the electrolyte. The activation energy of Li^+ transport was calculated based on equation 2:

$$\sigma = A \exp(-Ea / RT) \quad (2)$$

The activation energy of Li^+ desolvation was calculated based on equation 2:

$$1 / Rct = A \exp(-Ea / RT) \quad (3)$$

where A is the pre-exponential factor, E_a is the activation energy of Li^+ desolvation, R is the ideal gas constant, and T is the testing absolute temperature.

The Li^+ transference number (t_{Li}^+) was estimated by the electrochemical polarization method using Li||Li symmetric cell and was calculated by equation 3:

$$t_{Li+}=Is(\Delta V-I_oR_o)/ \quad I_o(\Delta V-I_sR_s)$$

(4)

where I_o and I_s are the initial and steady currents during polarization, R_o and R_s are the resistance values before and after the polarization, and ΔV (10 mV) is the constant applied voltage during polarization.

The exchange current density (J_0) of Li plating/stripping processes was determined by Tafel fitting of LSV results from −0. 2 V to 0.2 V with a scanning rate of 0.1 mV s^{-1} of Li symmetric cells.

Testing protocol: first plating 5 mAh cm^{-2} capacity and subsequently stripping the deposits until the cell voltage reaches 1 V vs. Li^+/Li, followed by three steps in order: a plating process at 5 mAh cm^{-2} (Q_1), ten stripping/plating cycles at 1 mAh cm^{-2} (Q_2) and a stripping process terminated at a cutoff voltage of 1 V with stripping areal capacity of Q_3. The average CE is calculated by the following equation:

$$CE = [(Q_3+9Q_2) / (Q_1+9Q_2)] * 100\%$$

(5)

Section S5. Computational Methods and Calculation

Computational Methods: MD simulations of two electrolyte systems were performed using Lammps with the all-atom optimized potentials for liquid simulations (pcff) force field. Different electrolyte models were constructed with LiTFSI, G_4, TTE and

PTEGDMA. The initial simulation boxes of dimensions. 71×86×55 Å3. Their structures were first relaxed by energy minimizing calculations, and then underwent an annealing from 0 to 298.15 K with the time step of 1 ps during 1 ns to reach the equilibrium state. Velocity-rescale thermostat with relaxation constant of 1 ps was used to control the temperature at 298.15 K. Berendsen's barostat with isothermal compressibility constant of 4.5×10^{-5} was used to control the pressure at 1.01325×10^{5} Pa. Periodic boundary conditions were applied in all directions. Particle-mesh Ewald method with cut-off distance of 10 Å was applied to treat the electrostatic interactions and the van der Waals forces. Upon quasi-equilibrium of the system, MD simulation for a total simulation time of 10 ns was performed at constant NVT ensemble, and the trajectory was saved every 10 ps. The coordination number was calculated based on the integral of the radial distribution function, and the total coordination number was set to 6.

Calculation: The binding energy was calculated as follows:

$$E=E_{ab}-E_a-E_b \quad (6)$$

Where E is the total energy of the adsorbed system, E_a and E_b represent the total energy of free species and bare surface, respectively.

Section S6. Supplementary Figures and Tables

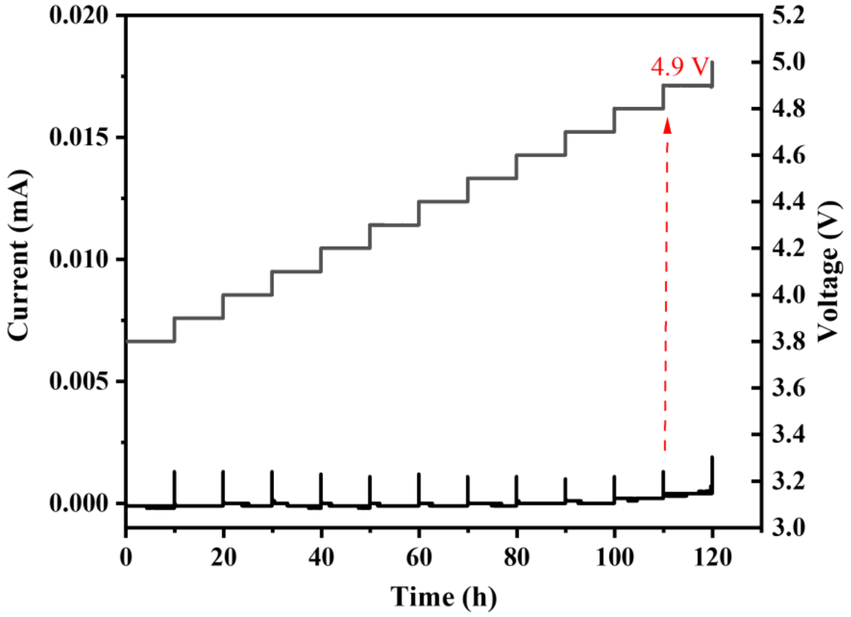

Figure S1. EFA curve of LHCE-GPE.

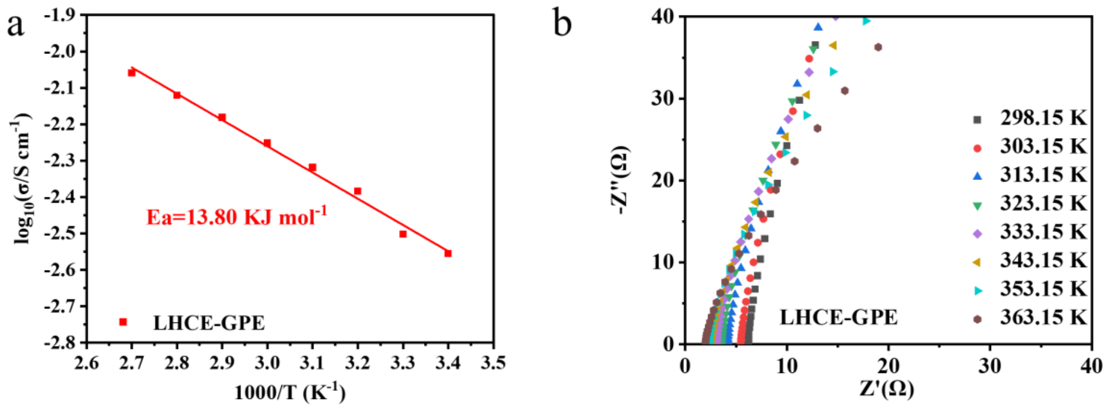

Figure S2. (a) Temperature-dependent ionic conductivities of LHCE-GPE. (b) EIS spectra of symmetric stainless steel cell using LHCE-GPE at different temperature.

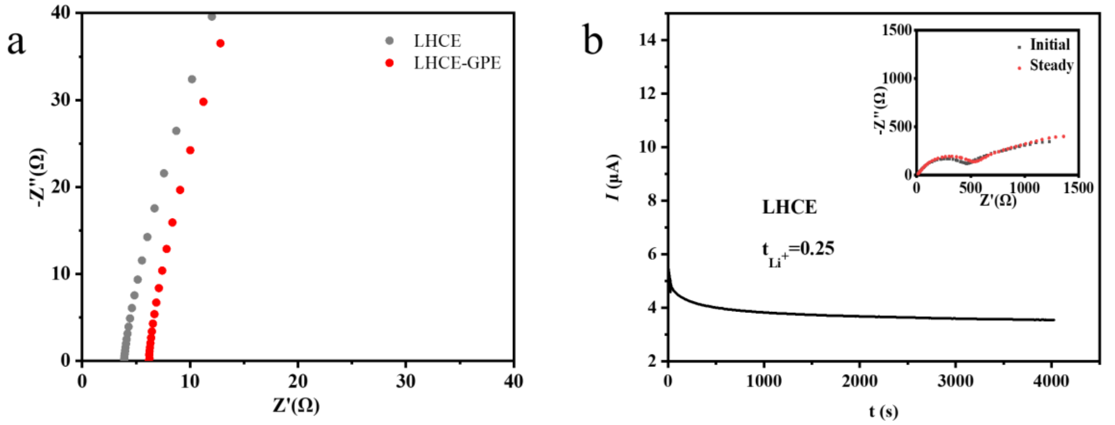

Figure S3. (a) Ionic conductivities of LHCE-GPE and LHCE. (b) Chronoamperometric and Nyquist plots (inset) of LHCE at 25 °C.

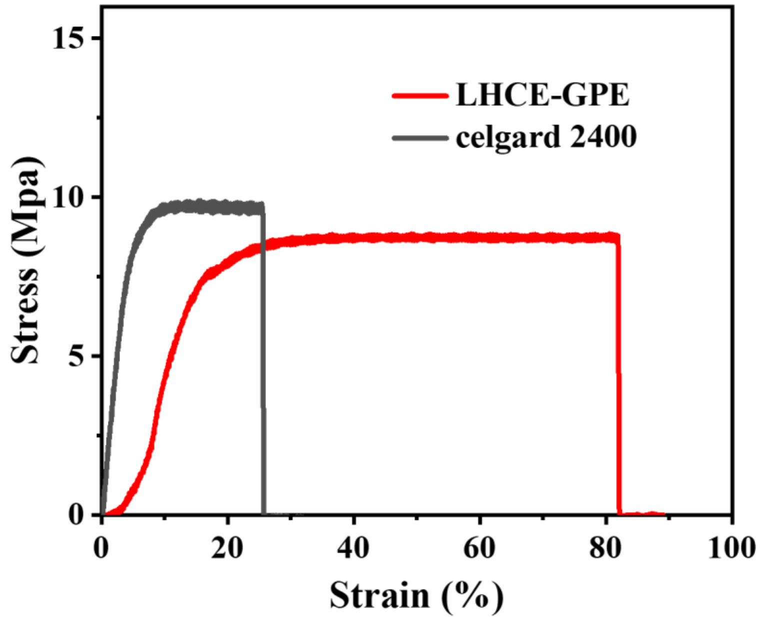

Figure S4. Stress-strain curves of LHCE-GPE and the Celgard 2400 separator.

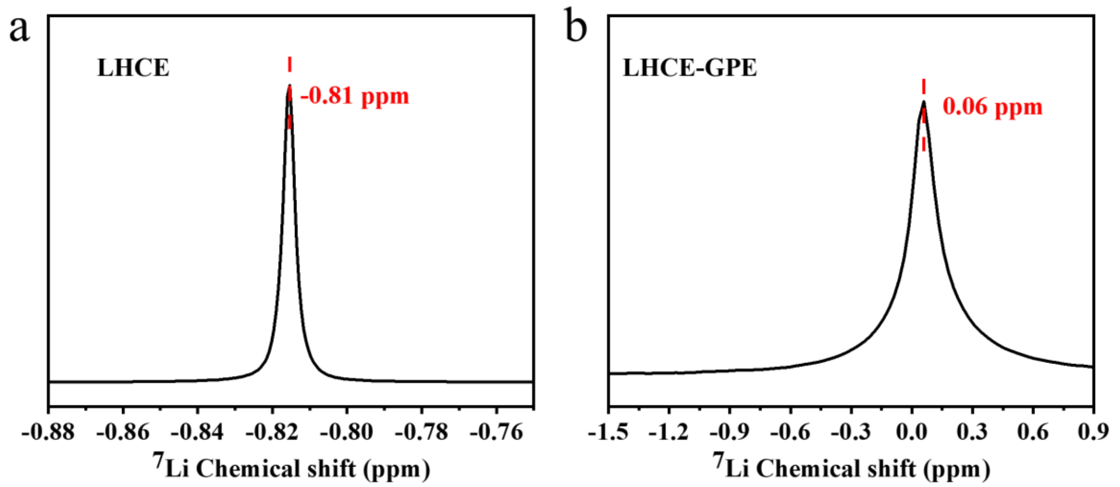

Figure S5. (a)The liquid ^{7}Li-NMR result of LHCE. (b) The solid-state ^{7}Li-NMR result LHCE-GPE.

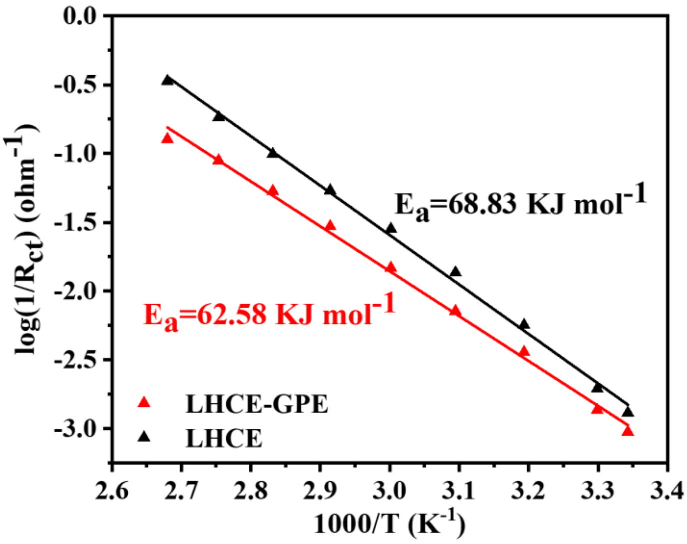

Figure S6. Arrhenius behavior of Li^+ desolvation in LHCE and LHCE-GPE.

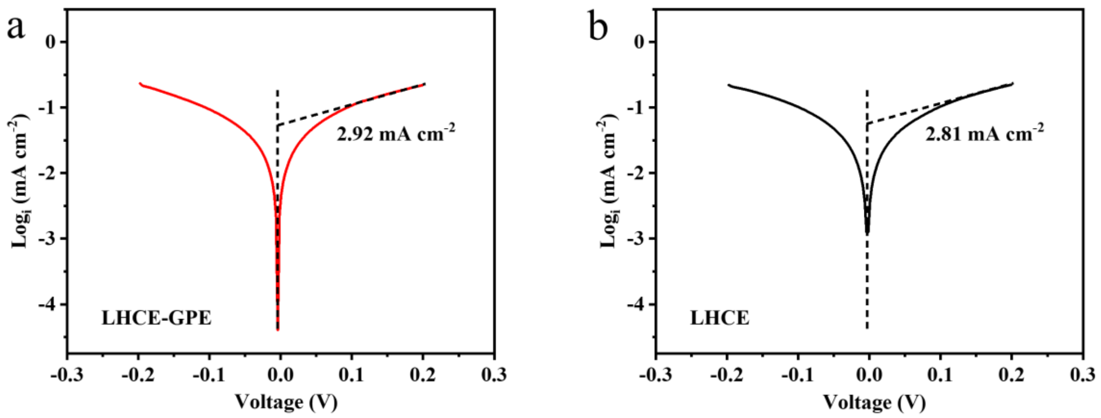

Figure S7. The exchange current density of Li plating/stripping processes was determined by Tafel fitting of LSV results from −0.2 V to 0.2 V with a scanning rate of 0.1 mV/s of Li symmetric cells using LHCE-GPE (a) and LHCE (b).

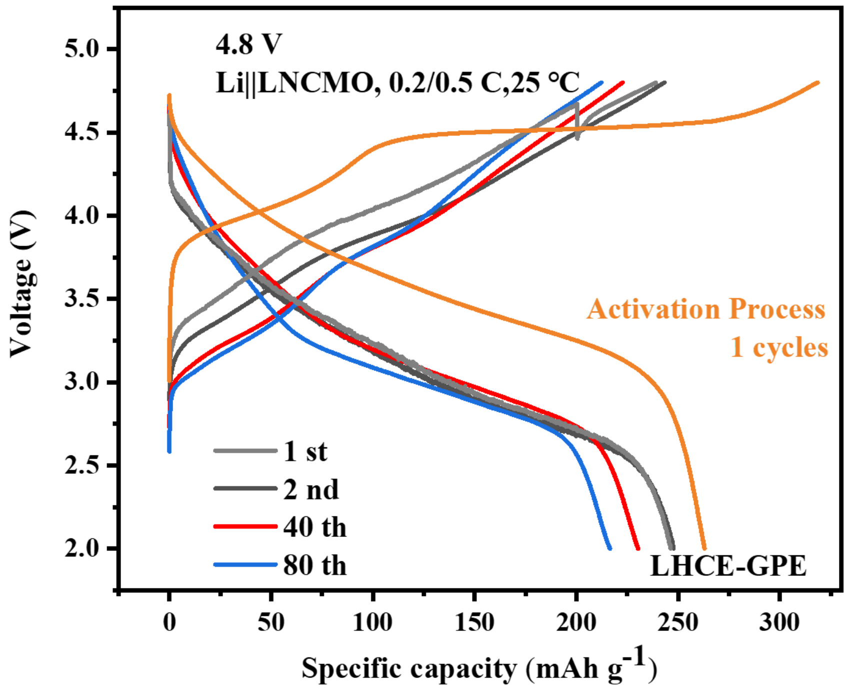

Figure S8. Voltage profiles of Li||LNCMO using LHCE-GPE at a high charge cut-off voltage of 4.8 V (The orange curve represents the activation of the first circle).

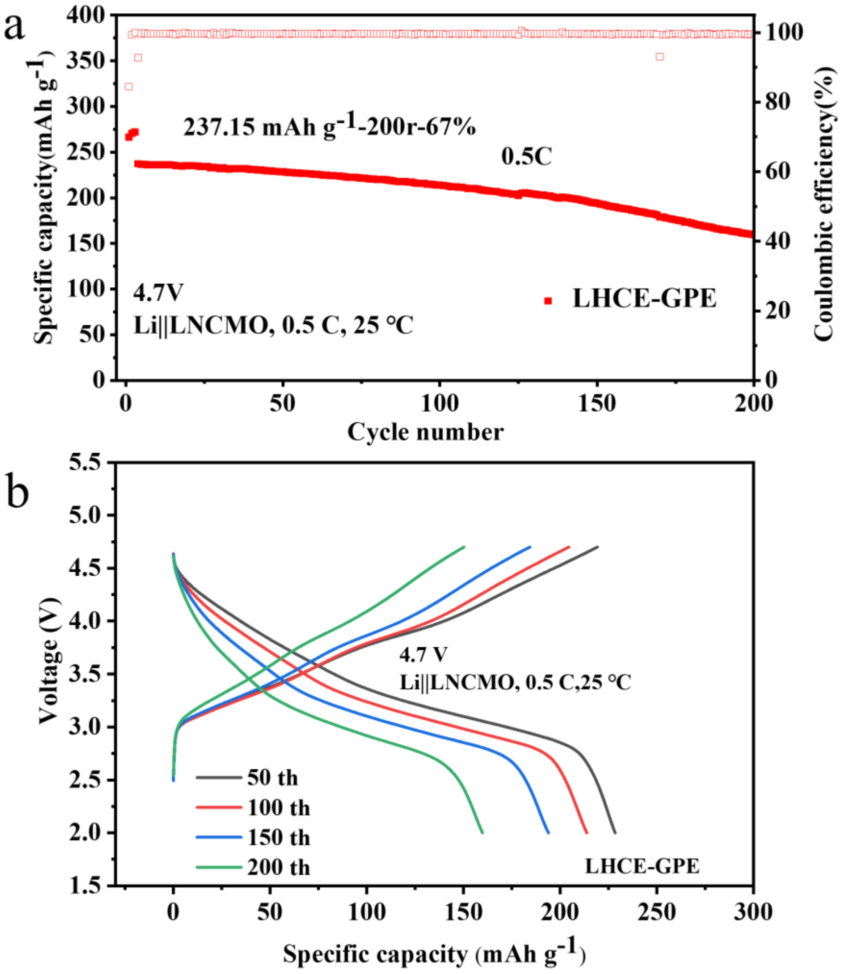

Figure S9. Cycling performance (a) and voltage profiles (b) at 0.5 C of 4.7 V Li||LNCMO using LHCE-GPE.

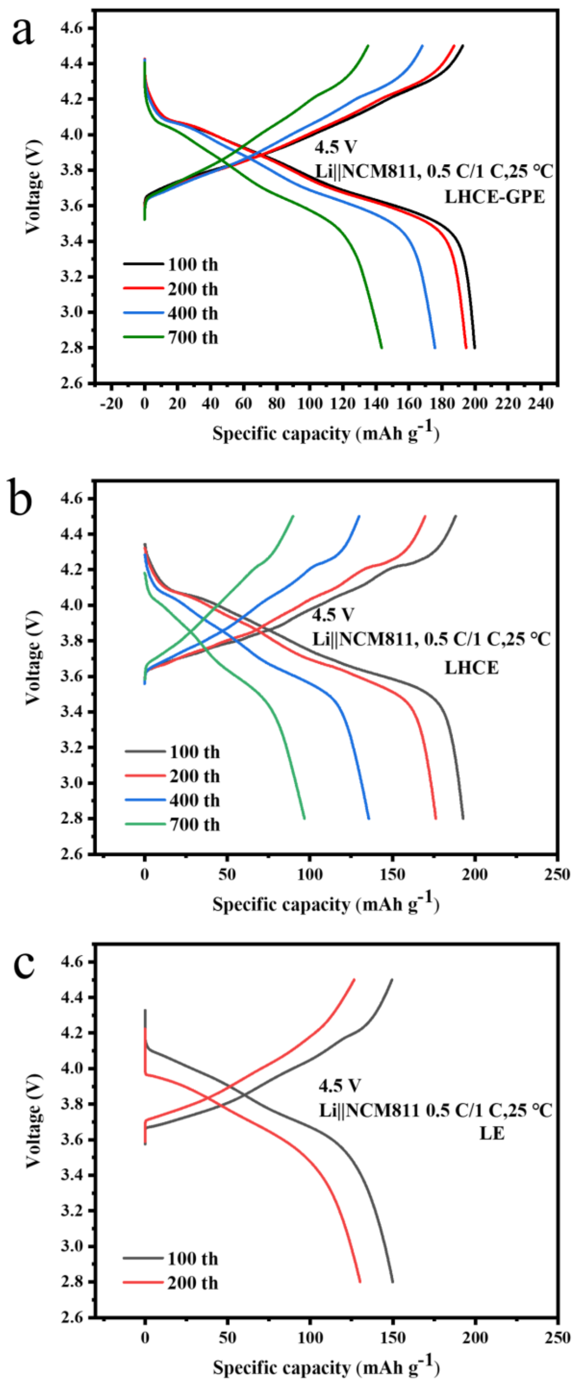

Figure S10. Voltage profiles of (a) Li|LHCE-GPE|NCM811, (b) Li|LHCE|NCM811 and (c) Li|LE|NCM811 batteries at 4.5 V.

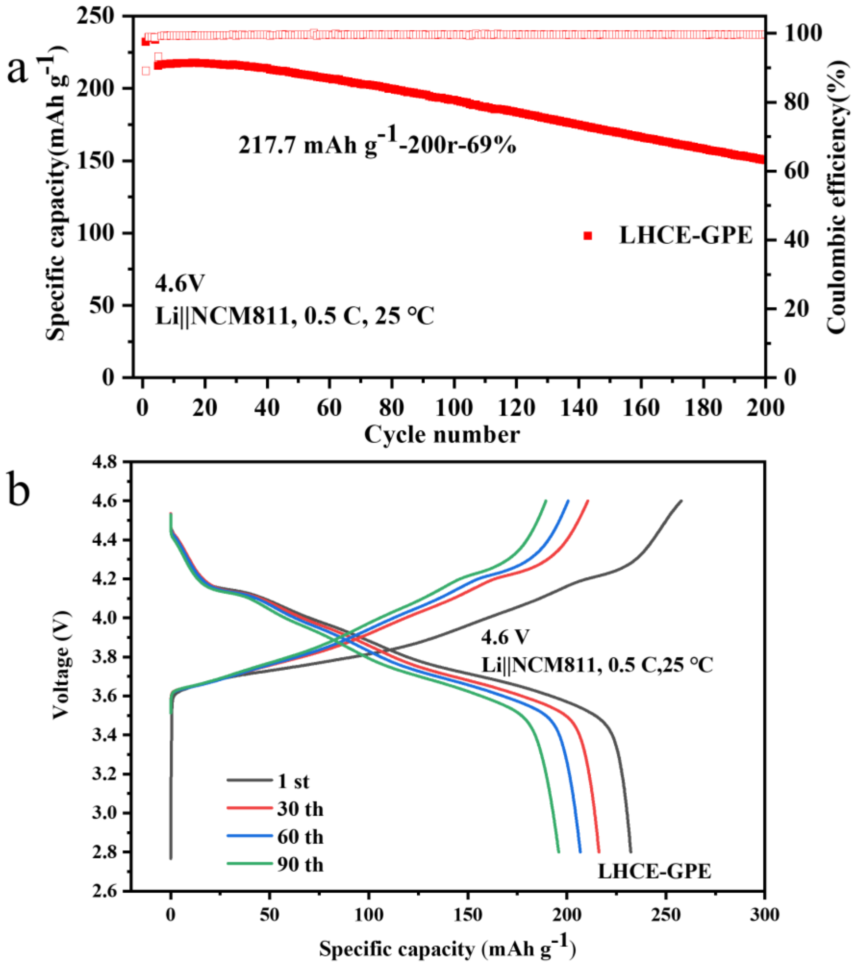

Figure S11. Cycling performance and corresponding voltage profiles at 0.5 C of 4.6 V Li||NCM811 using LHCE-GPE.

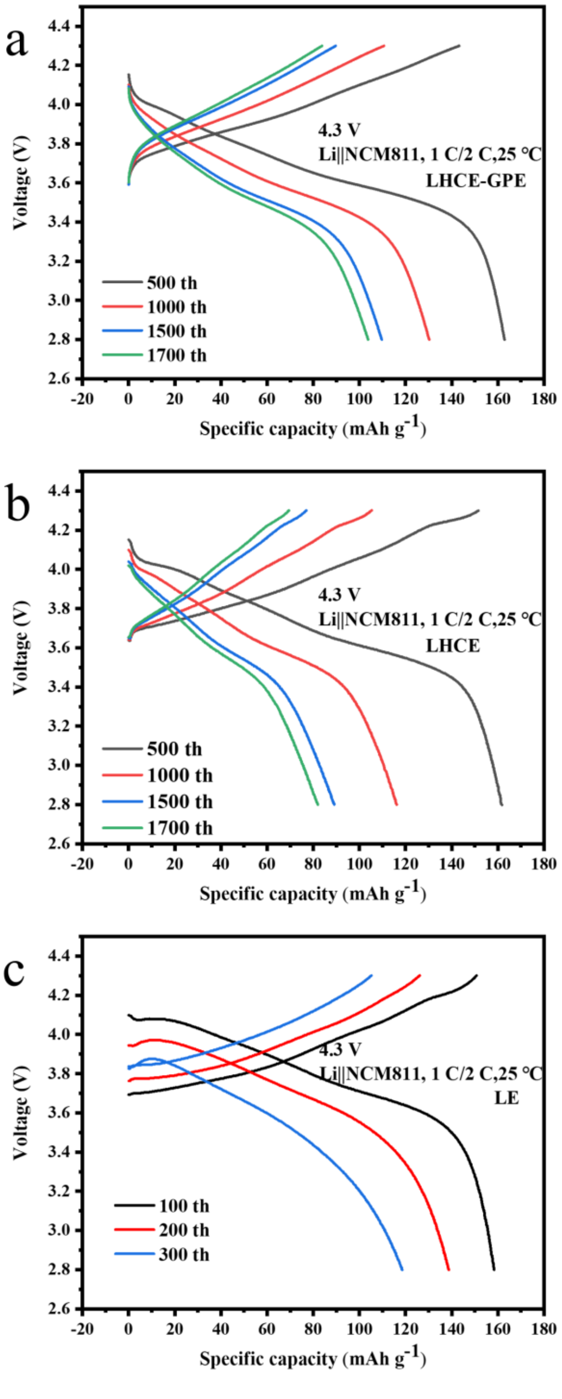

Figure S12. Voltage profiles of (a) Li|LHCE-GPE|NCM811, (b) Li|LHCE|NCM811 and (c) Li|LE|NCM811 batteries at 4.3 V.

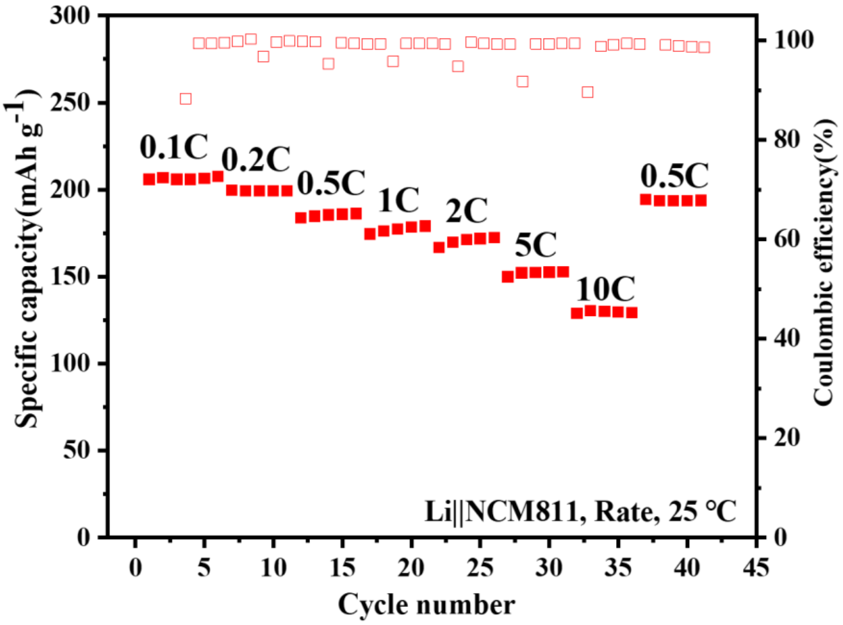

Figure S13. Rate performance of Li||NCM811 batteries with LHCE-GPE within 2.8-4.3 V.

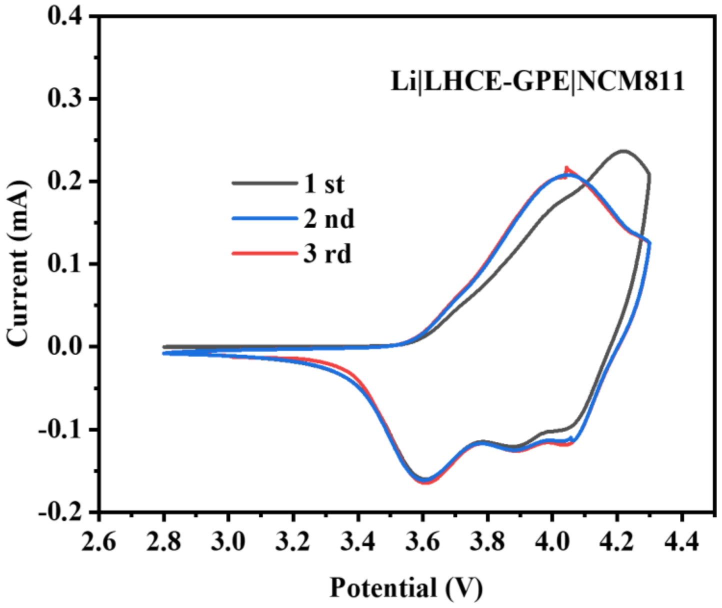

Figure S14. CV curves of the Li |LHCE-GPE|NCM811 cells with a scanning rate of 0.1 mV s^{-1} from 2.8 to 4.3 V.

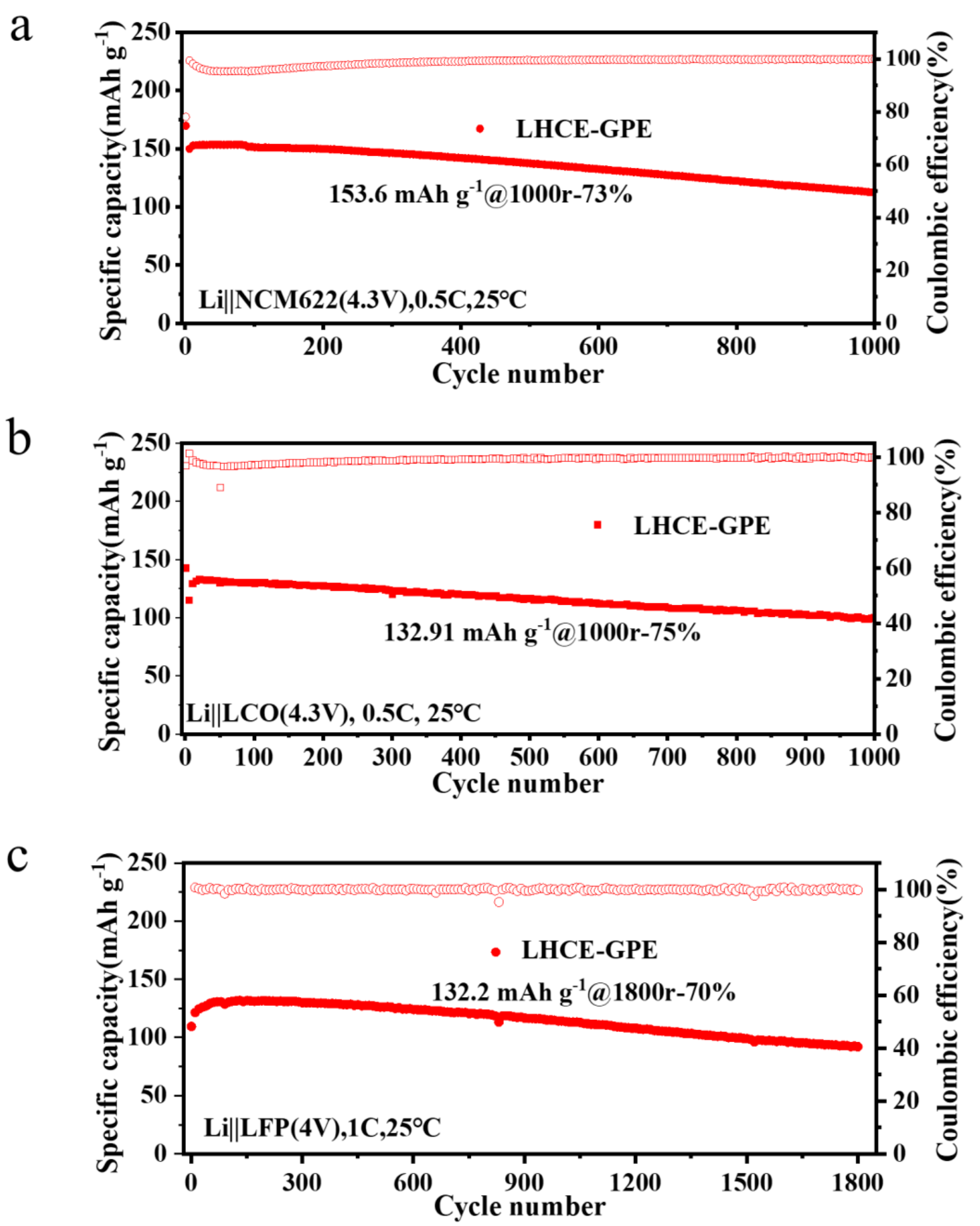

Figure S15. Cycling performances of Li||NCM622, Li||LCO and Li||LFP with LHCE-GPE.

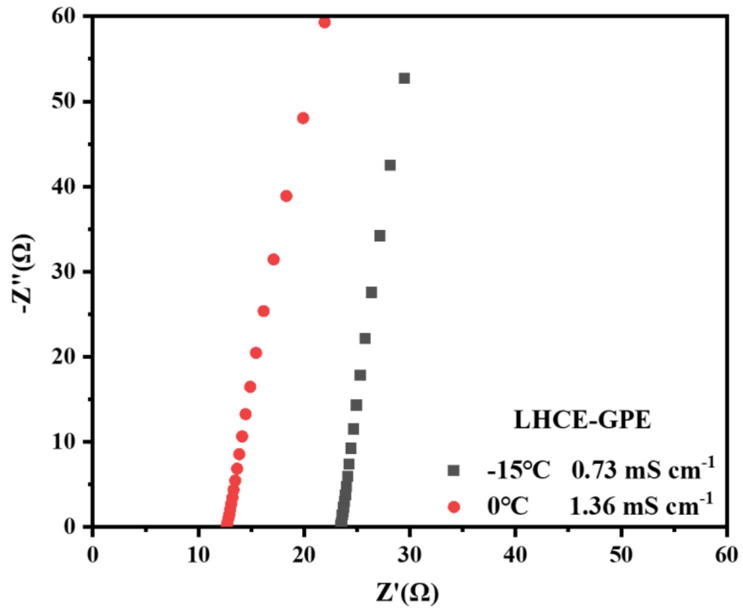

Figure S16. Ionic conductivities of LHCE-GPE at -15 °C and 0 °C.

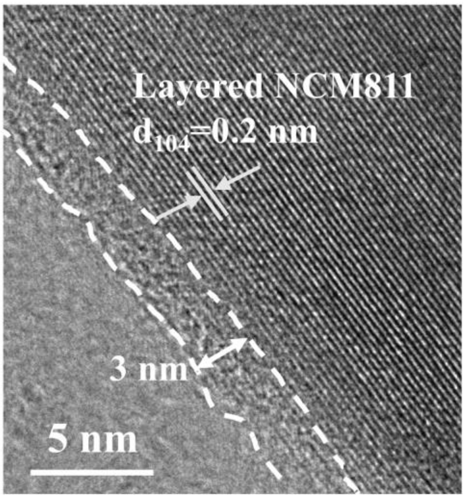

Figure S17. TEM image of NCM811 cathodes after 100 cycles with LHCE-GPE.

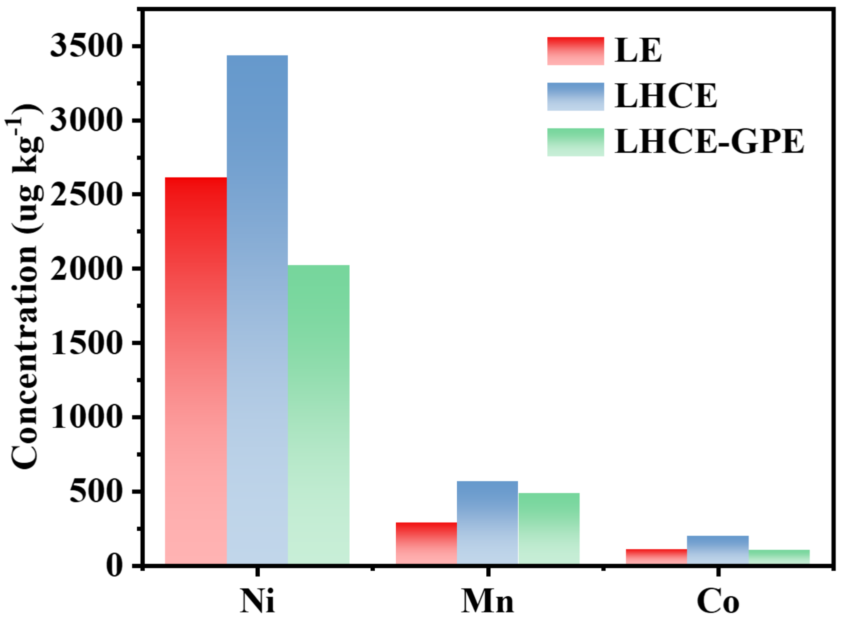

Figure S18. The amount of TM deposited on the cycled-Li metal anode after 30 cycles estimated from ICP-MS.

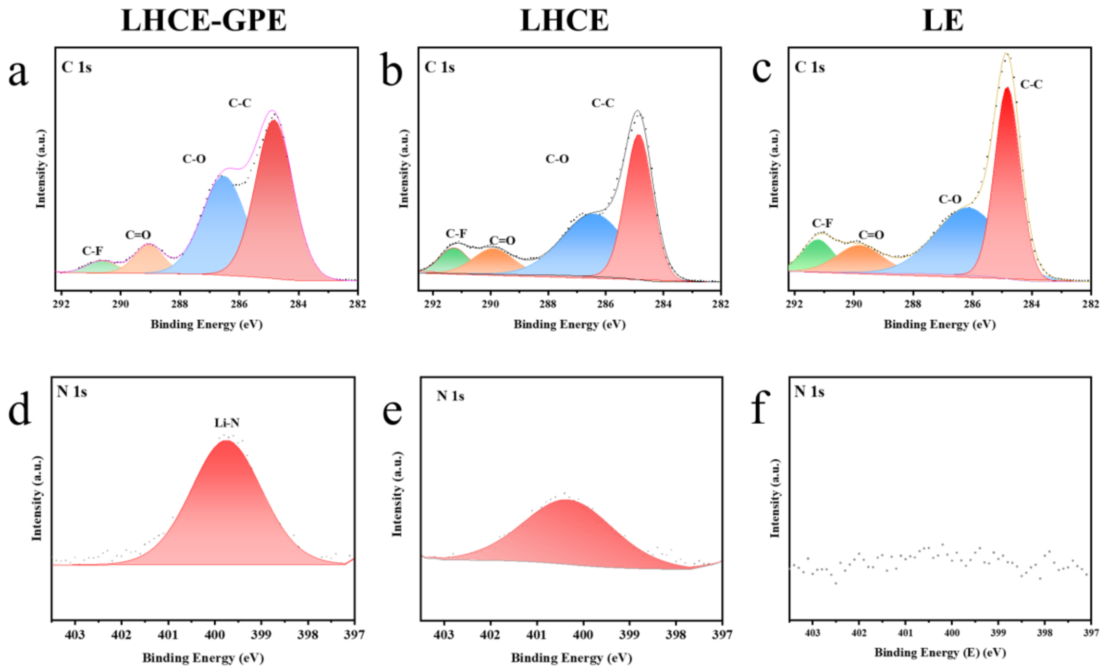

Figure S19. XPS spectra of C 1s, N 1s for NCM811 cathodes after 100 cycles under a cut-off voltage of 4.3 V using LHCE-GPE (a,d), using LHCE (b,e) , using LE (c,f).

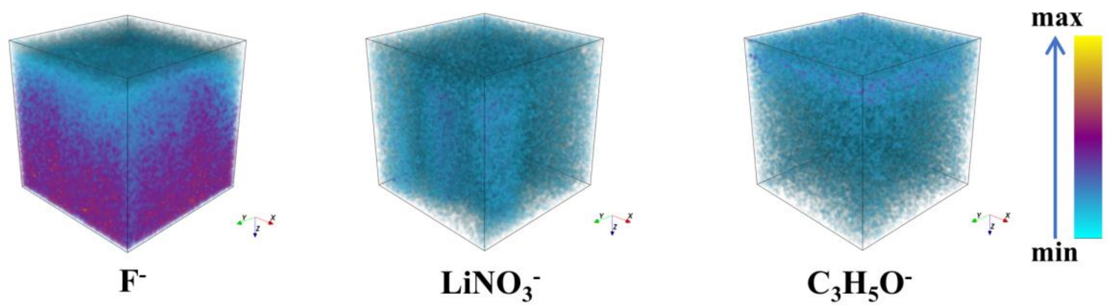

Figure S20. TOF-SIMS 3D mapping images of F^-, $LiNO_3^-$ and $C_3H_5O^-$ fragments obtained from NCM811 cathodes after cycling in LHCE-GPE.

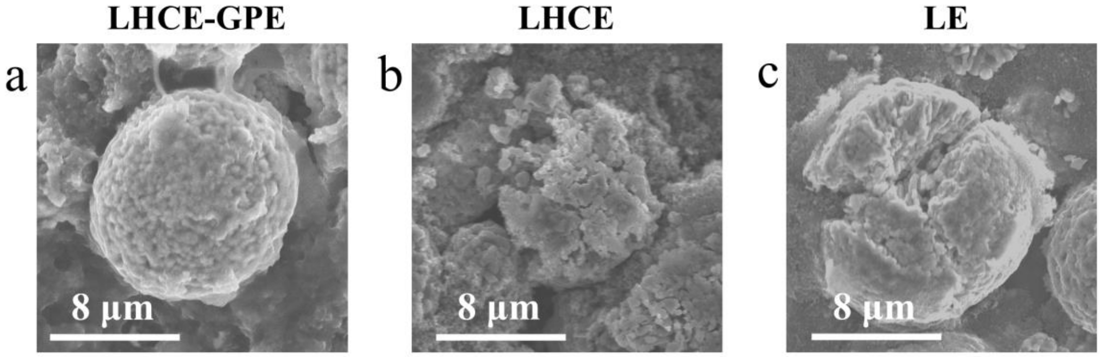

Figure S21. SEM images of NCM811 cathodes after 100 cycles with LHCE-GPE (a), with LHCE (b), and with LE (c).

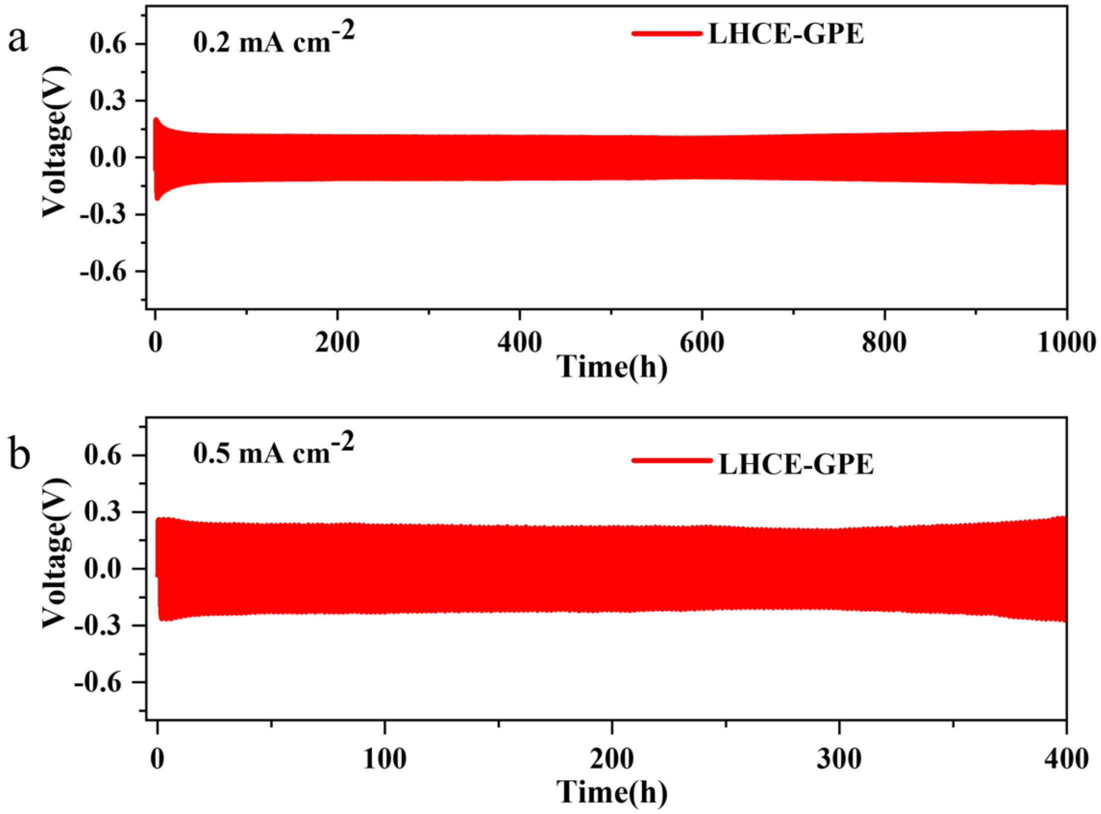

Figure S22. Polarization tests of Li||Li symmetric cells with LHCE-GPE at 0.2 mA cm^{-2} (a) and 0.5 mA cm^{-2} (b).

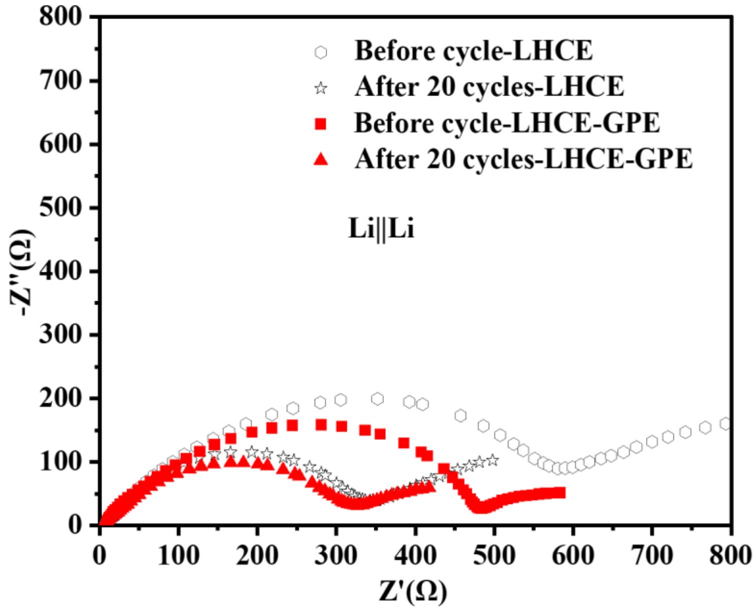

Figure S23. EIS spectra of Li||Li cells after different cycles.

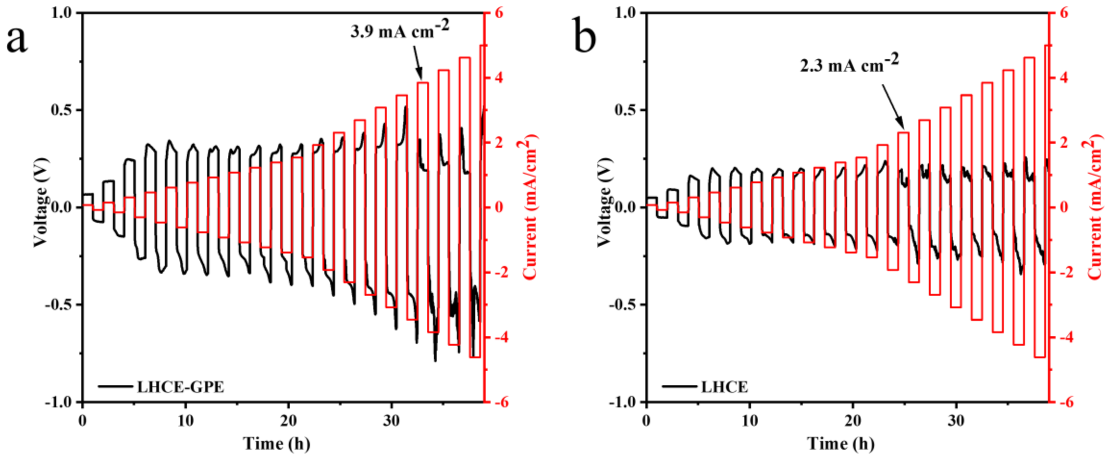

Figure S24. Critical current density tests of the symmetric Li||Li cell with LHCE-GPE (a) and LHCE (b).

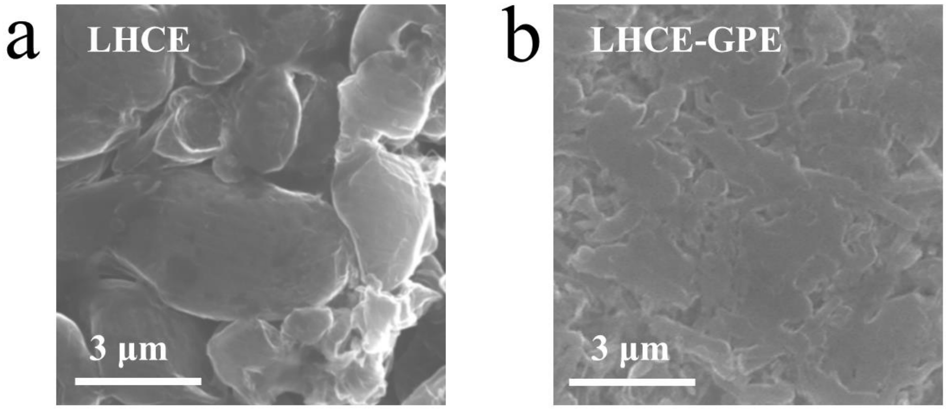

Figure S25. Li metal deposition morphologies in LHCE (a) and LHCE-GPE (b) in Li-Cu cells.

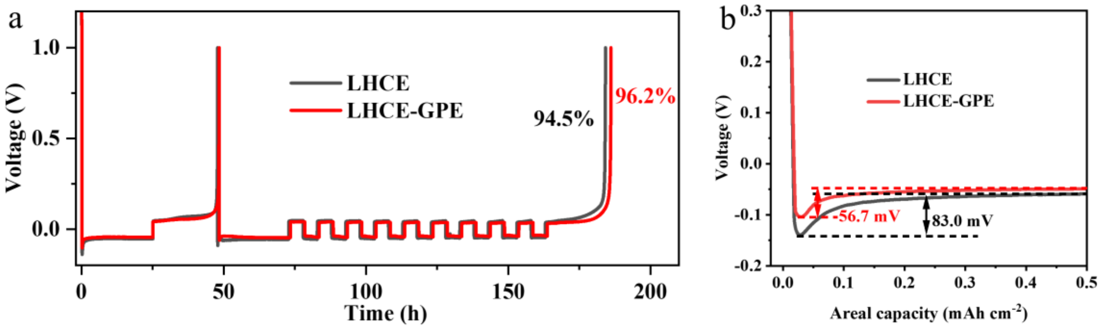

Figure S26. (a) CE results of Li||Cu cells tested using the modified Aurbach method. (b) The initial Li plating profiles of Figure S26 (a).

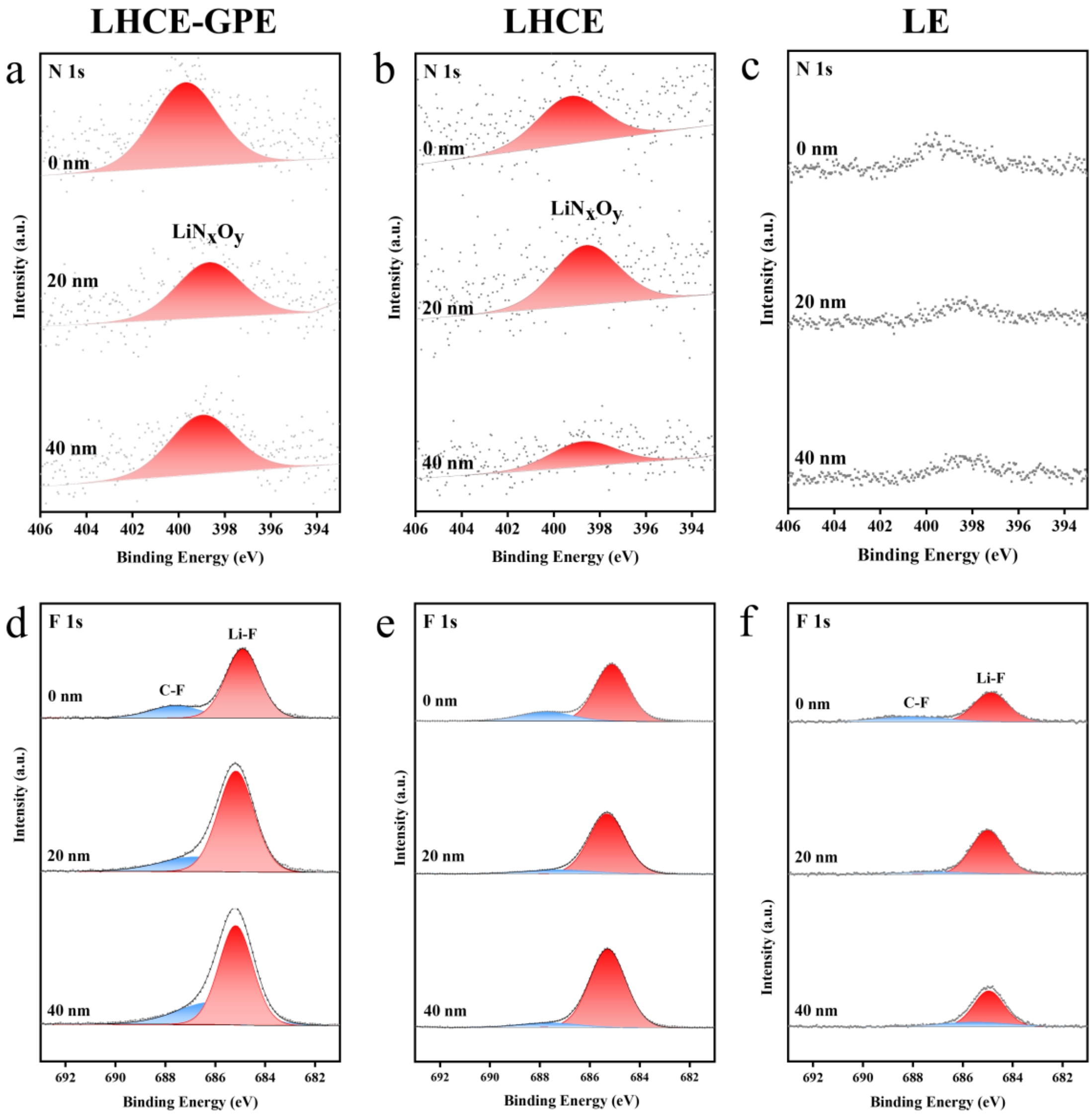

Figure S27. XPS depth profiles of N 1s, F 1s for Li anodes after 50 cycles under a cut-off voltage of 4.3 V using LHCE-GPE (a, d), LHCE (b, e), LE (c, f).

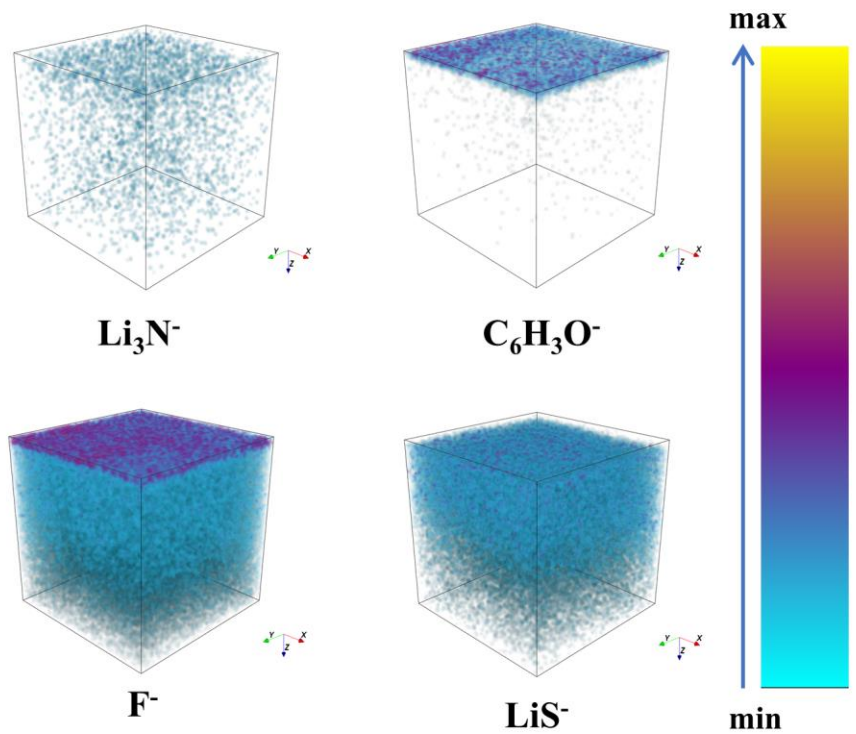

Figure S28. TOF-SIMS 3D mapping images of Li_3N^-, $C_6H_3O^-$, F^- and LiS^- fragments obtained from NCM811 cathodes after cycling in LHCE-GPE.

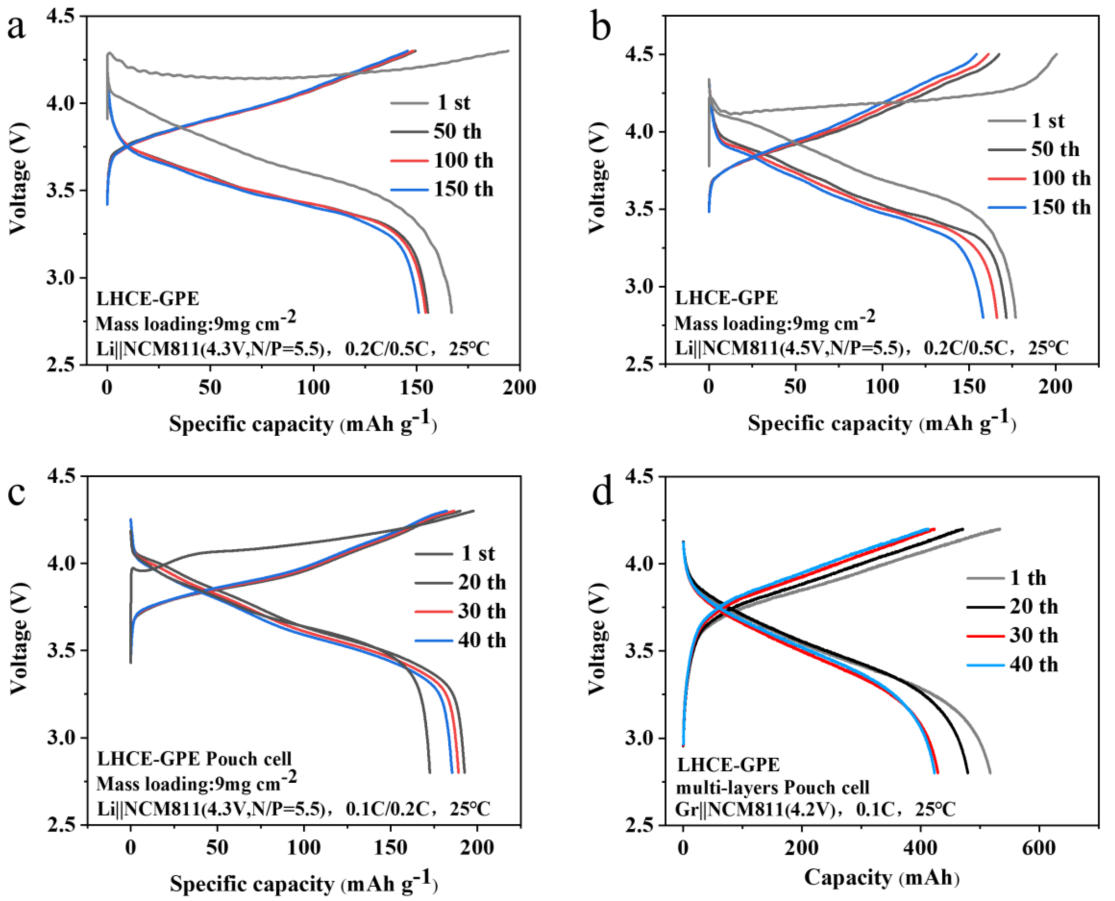

Figure S29. Voltage profiles for various configurations of lithium cells with LHCE-GPE electrolyte: (a) Coin-type full cell with Li|LHCE-GPE|NCM811 at 4.3 V, (b) the same at 4.5 V, (c) Single-layer Li|LHCE-GPE|NCM811 pouch cell at 4.3 V, and (d) Multi-layer Gr|LHCE-GPE|NCM811 pouch cell configuration at 4.2 V.

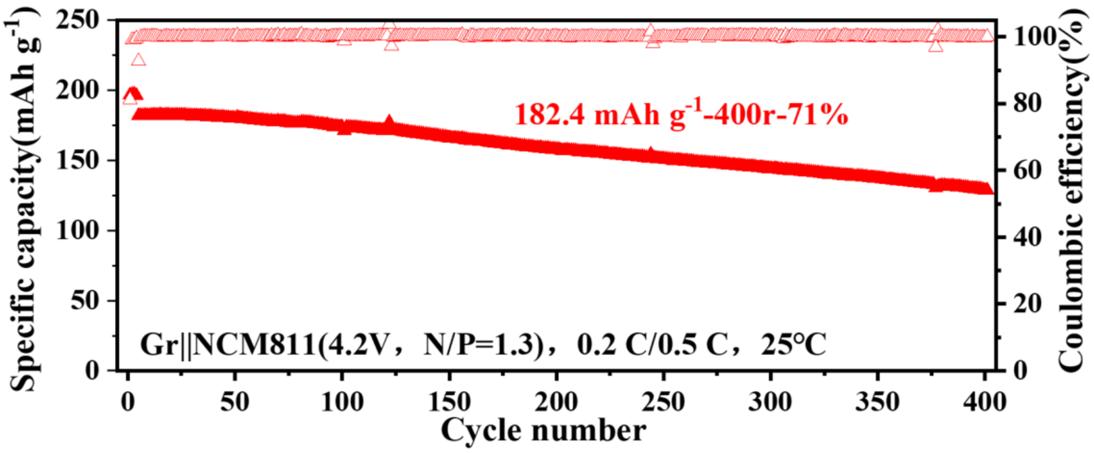

Figure S30. Cycling performance of 4.2 V Gr||NCM811 full cells using LHCE-GPE with N/P=1.3:1.

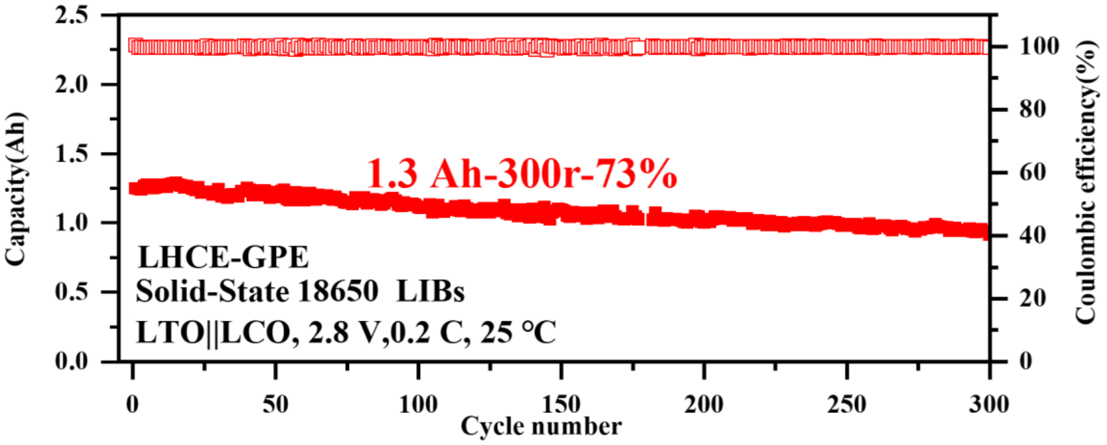

Figure S31. Cycling performance of 1.3 Ah Solid-state 18650 cylindrical $Li_4Ti_5O_{12}$||$LiMn_2O_4$ lithium-ion batteries.

Table S1: Li|| NCM811 18650 cylindrical cell parameters.

	Specifations	Parameters	
Cathode	Cathode material	$LiNi_{0.8}Co_{0.1}Mn_{0.1}O_2$	
	Areal loading (each side, mg cm^{-2})	8	
Anode	Anode material	Li	
	Thickness(each side, μm)	20	
Electrolyte	LHCE-GPE (g)	6	
Separator	16 μm PE		
Cell	N/P	2.58	
	E/C (g Ah^{-1})	6	
	Weight without package (g)	17.14	
	Total weight (g)	24.55	
	Areal capacity (mAh cm^{-2})	1.6	
	Voltage range (V)	2.8-4.3	2.8-4.7
	Discharge current (A)	0.1	0.1
	Average voltage (V)	3.77	3.91
	Discharge capacity (Ah)	0.9	1.1
	Energy capacity (Wh)	3.4	4.3
	Energy density (Wh kg^{-1}) (with package)	139	174
	Energy density (Wh kg^{-1}) (without package)	198	250

Table S2: Energy density parameters of different type of batteries.

Battery material	Li‖NCM811	Li‖NCM811	Si/C‖NCM811
Type of batteries	Pouch Cell	Cylindrical cell	
Capacity	0.9Ah (4.3V)	0.9 Ah (4.3 V)	3.28 Ah (4.6 V)
		1.1 Ah (4.7 V)	
Energy density	173 Wh kg^{-1} (4.3V)	139 Wh kg^{-1} (4.3V)	239 Wh kg^{-1} (4.6V)
		174 Wh kg^{-1} (4.7V)	
Energy density (without package)	---	198 Wh kg^{-1} (4.3V)	283 Wh kg^{-1} (4.6V)
		250 Wh kg^{-1} (4.7V)	

Table S3: Comparison of the cycling performance of polymer electrolyte-based Li||NCM811 full batteries with high loading at different cutoff voltages.

Polymer	Cathode material	Cutoff voltage	Rate	Specific capacity (mAh g-1)	Cycle numbers	Capacity Retention (%)	Ref
LiTFSI+G_4+TTE +PTEGDMA	50 μm Li<br>9 mg cm^{-2}	4.5	0.5C	174	200	85%	This work
		4.3	0.5	156	400	77%	
LiDFOB+LiTFSI + PVC	8 mg cm^{-2}	4.5 (60℃)	0.1C	168.7	200	85%	[1]
LiTFSI+LiDFOB +PEGDE	50 μm Li<br>9 mg cm^{-2}	4.5	0.1C	183.4	250	88.5	[2]
		4.3	0.1C	170	250	95.9	
LiTFSI +CA+SN+ PEGDMA	7 mg cm^{-2}	4.5	---	208	50	75.5	[3]
	10 mg cm^{-2}	4.4	---	178	100	82.5	
	6 mg cm^{-2}	4.3	0.05C	171	150	85.2	
$LiPF_6$ EC/DEC+PAN	25 μm Li<br>5.5 mg cm^{-2}	4.4	1C	200	100	78%	[4]
LiTFSI+DEE+SFE +PETEA+PBCPN	50 μm Li<br>20 mg cm^{-2}	4.4	0.5	170	200	81%	[5]
$LiPF_6$+EC-DEC+Poly(AA+2-EHA+PEGDMA)	5 mg cm^{-2}	4.3	0.5C	174.6	200	71%	[6]
LiTFSI/EMIMTFSI+ PAN+MXene-SiO_2	7 mg cm^{-2}	4.3	0.1C	165	50	72%	[7]

Table S4: parameters of Solid-state 18650 cylindrical $Li_4Ti_5O_{12}$| |$LiMn_2O_4$ lithium-ion batteries.

	Specifations	Parameters
Cathode	Cathode material	65% $LiCoO_2$ + 35% $LiMn_2O_4$
	Areal loading (each side, mg cm^{-2})	16.69
	Compact density (g ml^{-1})	3.29
	Thickness (each side, μm)	50.5
Anode	Anode material	$Li_4Ti_5O_{12}$
	Areal loading (each side, mg cm^{-2})	14.25
	Compact density (g ml^{-1})	1.95
	Thickness (each side, μm)	73
Separator	16 μm PE	
Cell	N/P	1.1
	Voltage range (V)	1.5-2.8

Supplementary references

1. Wang C, Zhao X, Li D *et al.* Anion-modulated Ion Conductor with Chain Conformational Transformation for Enhanced Interfacial Phase Stabilization of High-Voltage Lithium Metal Batteries. *Angew Chem Int Ed* 2024; **63**: e202317856.
2. Zhang M, Wang H, Shao A *et al.* Enabling 4.5 V Solid Polymer Batteries through a 10 µm, Crosslinked Polyether Electrolyte. *Adv Energy Mater* 2024; **14**: 2303932.
3. Gong Y, Wang C, Xin M *et al.* Ultra-thin and high-voltage-stable Bi-phasic solid polymer electrolytes for high-energy-density Li metal batteries. *Nano Energy* 2024; **119**: 109054.
4. Zhou S, Fu C, Chang Z *et al.* Conductivity gradient modulator induced highly reversible Li anodes in carbonate electrolytes for high-voltage lithium-metal batteries. *Energy Storage Mater* 2022; **47**: 482-490.
5. Meng Y, Zhou D, Liu R *et al.* Designing phosphazene-derivative electrolyte matrices to enable high-voltage lithium metal batteries for extreme working conditions. *Nat Energy* 2023; **8**: 1023–1033.
6. Gao S, Pan Y, Li B *et al.* Ultra-Stretchable, Ionic Conducting, Pressure-Sensitive Adhesive with Dual Role for Stable Li-Metal Batteries. *Adv Funct Mater* 2022; **33**: 2210543.
7. Liu Q, Dan Y, Kong M *et al.* Sandwich-Structured Quasi-Solid Polymer Electrolyte Enables High-Capacity, Long-Cycling, and Dendrite-Free Lithium Metal Battery at Room Temperature. *Small* 2023; **19**: 2300118.
